# Supplementary material for: Mechanochemically accessing a challenging-to-synthesize depolymerizable polymer
Source: Nat Commun. 2023 Jan 14;14:225. doi: 10.1038/s41467-023-35925-2 (PMC9840636; doi:10.1038/s41467-023-35925-2)
Supplement: Supplementary file 1 — Supplementary Information [file 41467_2023_35925_MOESM1_ESM.pdf]

Supplementary Information

Mechanochemically accessing a challenging-to-synthesize depolymerizable polymer

Tze-Gang Hsu,[‡1] Shiqi Liu,[‡1] Xin Guan,[1] Seiyoung Yoon,[1] Junfeng Zhou,[1] Wei-Yuan Chen,[2] Sanjay Gaire,[2] Joshua Seylar,[1] Hanlin Chen,[1] Zeyu Wang,[1] Jared Rivera,[1] Leyao Wu,[1] Christopher J. Ziegler,[2] Ruel McKenzie,[1] and Junpeng Wang*[1]

[1]School of Polymer Science and Polymer Engineering, The University of Akron, 170 University Ave, Akron, Ohio 44325, United States.
E-mail: jwang6@uakron.edu
[2]Department of Chemistry, The University of Akron, 170 University Ave, Akron, Ohio 44325, United States.
[‡]These authors contributed equally: Tze-Gang Hsu, Shiqi Liu.

Table of Contents

I. General Procedures 3

Materials... 3

Instruments 3

II. Small Molecule Synthesis 4

Synthesis of DHF dimer 4

Alternative Synthesis of ((1R,5S,6R,7S)-3-oxabicyclo[3.2.0]heptane-6,7-diyl)dimethanol (1) 4

Synthesis of M1 7

Synthesis of Biscyclooctene Ester Crosslinker 8

III. Polymer Synthesis 9

Synthesis of P1... 9

Synthesis of P2.. 10

Synthesis of PDHF (from DHF) 10

Synthesis of PDHF (from DHF dimer) 12

IV. Thermal Properties of Polymers and Networks 14

V. Sonication Experiments 15

General 15

Calculation of % Ring Opening of P1 from ^{1}H NMR 15

Calculation of Z/E Ratio for Mechanochemically Generated Olefins from P1 to SP2 16

Calculation of % Ring Opening of P2 from ^{1}H NMR 18

Calculation of Z/E Ratio for Mechanochemically Generated Olefins from P2 to SP2 19

Sonication Control Study for 5 kDa P1 20

Calculation of Scission Cycle from M_n Changes[5] 21

Extent of Ring Opening (ϕ) vs Scission Cycle 21

VI. Depolymerization Experiments 23

General 23
Calculation of Depolymerization Percentage 23
Depolymerization Results for 44 kDa SP1 24
Mass Spectrometry and ^{1}HNMR Results for P1 Reacting with G2 26
VII. Bulk Activation 28
Extrusion 28
Polymer Network PN1 Synthesis 29
Compressive Testing of PN1 30
Ball Milling 30
Depolymerization Study for Bulk Activation Sample 30
Calculation of % DHF in Depolymerization of Ball-Milled PN1 31
Degradation Study for Ball Milled Sample 32
Calculation of % Ring Opening in Degraded PN1 34
VIII. Mass Spectrometry 35
IX. X-Ray Crystallographic Study 39
X-Ray Crystallographic Study of M1 39
X. ^{1}H and ^{13}C NMR Spectra 42
XI. Optimized Geometries 52
XII. Supplementary References 58

I. General Procedures

Materials

Tetrahydrofuran (THF), *N*,*N*-dimethylformamide (DMF) were obtained from Sigma-Aldrich and dried by immersing in 4 Å molecular sieves desiccant (beads, 4-8 mesh, Sigma-Aldrich) overnight before use. $CDCl_3$ and DMSO-d_6 were purchased from Cambridge Isotope Laboratories. Other solvents were purchased from Fisher Chemical and used without further purification. All other chemicals were purchased from Sigma-Aldrich or Fisher Chemical without further purification before use unless specified.

Instruments

All GPC experiments were carried out using Tosoh EcoSEC HLC-8320 GPC with two 17393 TSKgel columns (7.8 mm ID x 30 cm, 13 μm) and one 17367-TSKgel Guard Column (7.5 mm ID x 7.5 cm, 13 μm) using preservative-free HPLC grade THF (obtained from Fisher Chemical) at a flow rate of 1 mL min^{-1} at 40°C. Purification by preparative GPC were done by LaboACE LC-5060 which was connected to two JAIGEL-2HR columns using HPLC grade chloroform with 0.75% ethanol as preservative (obtained from Fisher Chemical) at a flow rate of 10 mL min^{-1}. ^{1}H and ^{13}C NMR spectra were collected on a Varian 500 MHz spectrometer using $CDCl_3$ or DMSO-d_6 as the solvent and referenced to residual solvent peak (δ = 7.26 in ^{1}H, 77.16 in ^{13}C for $CDCl_3$; δ = 2.5 in ^{1}H, 39.52 in ^{13}C for DMSO-d6). X-ray crystallographic study was carried out using Bruker ApexII Duo with comounted Mo and Cu microfocus radiation sources and Quazar optics for increased source brightness. ESI-MS spectra were recorded on a Waters Synapt HDMS quadrupole/time-of-flight (Q-ToF) mass spectrometer (Waters, Beverly, MA) in positive ion mode. Samples were prepared to a final concentration of 1 μg mL^{-1} in methanol. Each sample was introduced to the electrospray source via direct infusion at a flow rate of 5 μL/min, with source parameters as follows: capillary voltage: 3.15 kV; cone voltage: 35 V; sampling cone voltage: 3.2 V; source temperature: 90 °C; desolvation temperature: 150 °C. High resolution-atmospheric pressure chemical ionization mass spectra (HR-APCI-MS) were acquired using a Waters Synapt HDMS Quadrupole/Time-of-Flight (Q-ToF) Mass Spectrometer (Waters, Beverly, MA) equipped with an atmospheric solids analyses probe (ASAP) in positive ion mode.

II. Small Molecule Synthesis

Synthesis of DHF dimer

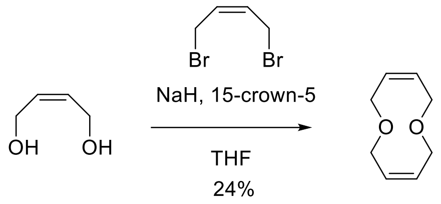

DHF dimer

(3Z,8Z)-2,5,7,10-tetrahydro-1,6-dioxecine (DHF dimer)

To a suspension of sodium hydride (powder, 90%, 1.2 g, 50 mmol, 5 equiv.) in dry THF (400 mL) was added *cis*-2-butene-1,4-diol (881 mg, 10 mmol,1 equiv.) and 15-crown-5 (4.4 g, 20 mmol, 2 equiv.), and the mixture was stirred for 1 h under N_2. *Cis*-1,4-dibromo-2-butene (2.13 g, 10 mmol, 1 equiv.) in THF (10 mL) and additional 490 mL THF were added to the reaction mixture. The reaction was allowed to stir for 1 day before it was quenched by adding several drops of water. After removal of THF on a rotavapor, the residue was purified by column chromatography (ethyl acetate/hexane = 1:2) to obtain DHF dimer as a white solid (340 mg; yield: 24%).

^{1}H NMR (500 MHz, $CDCl_3$, ppm) δ 5.87 – 5.71 (m, 4H), 4.26 – 4.08 (m, 8H).

^{13}C NMR (125 MHz, $CDCl_3$, ppm): δ 132.02, 59.98.

MS (ASAP) m/z: calcd for $C_8H_{13}O_2$ $[M+H]^+$, 141.0916; found 141.0938.

Alternative Synthesis of ((1R,5S,6R,7S)-3-oxabicyclo[3.2.0]heptane-6,7-diyl)dimethanol (1)

In addition to previously reported route,[1] a more efficient synthetic route was developed for the synthesis of **1.**

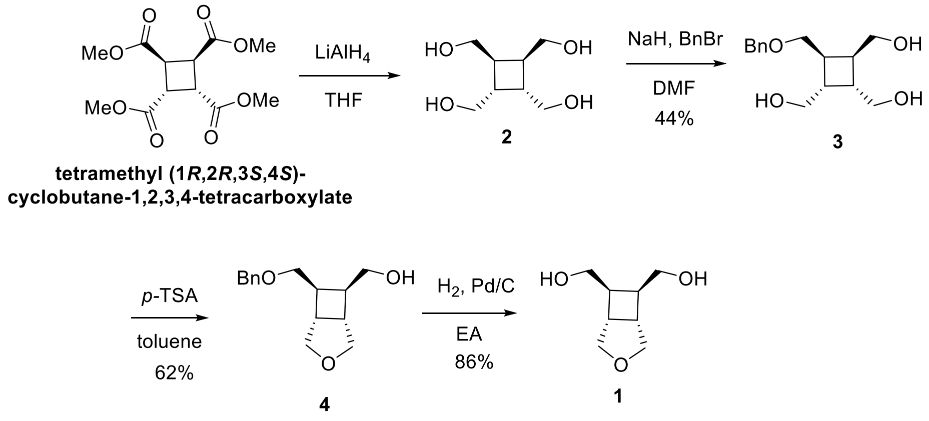

((1R,2R,3S,4S)-cyclobutane-1,2,3,4-tetrayl)tetramethanol (2)

2 was prepared according to a reported procedure.[2] To the commercially available tetramethyl (1R,2R,3S,4S)-cyclobutane-1,2,3,4-tetracarboxylate (300 mg, 1.04 mmol, 1.0 equiv.) in dry THF (10 mL) was added $LiAlH_4$ (158 mg, 4.16 mmol, 4.0 equiv.) portionwise at 0 °C, and the reaction was allowed to stir overnight at room temperature. The reaction was then quenched by sequentially adding H_2O (160 μL), 15 wt% $NaOH_{(aq)}$, and then H_2O (473 μL). Celite (800 mg) was poured into the mixture, and the mixture was stirred for another 15 min. The mixture was filtered and washed with MeOH (10 mL × 3), and the filtrate was collected. After removal of the solvent on a rotavapor, the residue was purified by column chromatography (MeOH/DCM =1/4) to obtain **2** as colorless oil (137 mg, yield: 75%).

^{1}H NMR (500 MHz, DMSO-d6, ppm): δ 4.50 (t, J = 5.1 Hz, 4H), 3.63 – 3.50 (m, 4H), 3.48 – 3.37 (m, 4H), 2.18 – 2.04 (m, 4H).

^{13}C NMR (125 MHz, DMSO-d_6, ppm): δ 61.32, 38.76.

MS (ESI) m/z: calcd for $C_8H_{16}O_4Na$ $[M+Na]^+$, 199.0940; found 199.0946.

((1S,2S,3S,4S)-4-((benzyloxy)methyl)cyclobutane-1,2,3-triyl)trimethanol (3)

To a vigorously stirred, 15 mL DMF solution of **2** (1 g, 5.67 mmol,1.0 equiv.) was added NaH (60% in oil, 150 mg, 6.24 mmol, 1.1 equiv.) in dry DMF (15 mL) under N_2 at 0 °C. After 40 min of stirring at room temperature, benzyl bromide (970 mg, 5.67 mmol,1.0 equiv.) was added quickly to the reaction mixture using a syringe, and the reaction was allowed to stir overnight. Vacuum distillation was applied to

remove DMF, and the residue was purified by column chromatography (MeOH/DCM = 1/12) to obtain **3** as colorless oil (664 mg, yield: 44%).

^{1}H NMR (500 MHz, $CDCl_3$, ppm) δ 7.42 – 7.27 (m, 5H), 4.54 (s, 2H), 3.90 – 3.77 (m, 2H), 3.77 – 3.68 (m, 2H), 3.68 – 3.61 (m, 2H), 3.58 – 3.46 (m, 2H), 2.88 (s, 3H), 2.47 – 2.21 (m, 4H).

^{13}C NMR (125 MHz, $CDCl_3$, ppm): δ 137.38, 128.73, 128.20, 128.10, 73.74, 70.07, 62.43, 62.39, 62.35, 38.46, 38.21, 38.05, 35.47.

MS (ESI) m/z: calcd for $C_{15}H_{22}O_4Na$ $[M+Na]^+$, 289.141; found 289.1400.

((1S,5S,6S,7S)-7-((benzyloxy)methyl)-3-oxabicyclo[3.2.0]heptan-6-yl)methanol (4)

To **3** (400 mg, 1.5 mmol,1.0 equiv.) in toluene (30 mL) was added *p*-toluenesulfonic acid monohydrate (14 mg, 0.075 mmol, 0.05 equiv.), and the reaction was heated to reflux using Dean-Stark apparatus for 16 hours. After removal of toluene on a rotavapor, the residue was purified by column chromatography (ethyl acetate/hexane = 3/1) to obtain **4** as colorless oil (231 mg, yield: 62%).

^{1}H NMR (500 MHz, $CDCl_3$, ppm) δ 7.39 – 7.24 (m, 5H), 4.53 (s, 2H), 3.86 (d, *J* = 9.0 Hz, 2H), 3.77 (dd, *J* = 11.6, 10.0 Hz, 1H), 3.73 (t, *J* = 10.0 Hz, 1H), 3.54 – 3.39 (m, 4H), 3.29 (s, 1H), 2.52 – 2.28 (m, 4H).

^{13}C NMR (125 MHz, $CDCl_3$, ppm): δ 137.27, 128.55, 128.53, 127.99, 127.94, 73.44, 73.43, 73.36, 70.00, 62.19, 41.16, 38.53, 38.45, 38.22.

MS (ESI) m/z: calcd for $C_{15}H_{20}O_3Na$ $[M+Na]^+$, 271.1300; found 271.1310.

((1R,5S,6R,7S)-3-oxabicyclo[3.2.0]heptane-6,7-diyl)dimethanol (1)

A mixture of 5 wt% Pd/C (10 mg), **4** (200 mg, 0.8 mmol) and ethyl acetate (8 mL) was charged into a pressure bottle settled on a hydrogenation apparatus (3920 Shaker Hydrogenation Apparatus, Parr Instrument Company). The reaction mixture was shaken at room temperature under 35 psi hydrogen pressure for 20 h. Pd/C was filtered over celite. After removal of the solvent on a rotavapor, the

residue was purified by flash column chromatography (ethyl acetate) to obtain **1** as colorless oil (109 mg, yield: 86%).

^{1}H NMR (500 MHz, $CDCl_3$, ppm) δ 4.29 (s, 2H), 3.81 (d, *J* = 9.4 Hz, 2H), 3.79 – 3.71 (m, 2H), 3.57 – 3.47 (m, 2H), 3.44 – 3.34 (m, 2H), 2.45 – 2.34 (m, 2H), 2.33 – 2.18 (m, 2H).

^{13}C NMR (125 MHz, $CDCl_3$, ppm): δ 73.43, 61.94, 40.70, 38.15.

MS (ESI) m/z: calcd for $C_8H_{14}O_3Na$ $[M+Na]^+$, 181.0840; found 181.0856.

Synthesis of M1

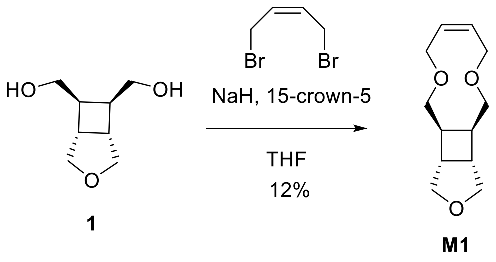

(3aR,3bS,11aR,11bS,Z)-1,3,3a,3b,4,6,9,11,11a,11b-decahydrofuro[3',4':3,4] cyclobuta[1,2-c][1,6]dioxecine (M1)

To a suspension of sodium hydride (powder, 90%, 228 mg, 9.48 mmol, 5 equiv.) in dry THF (100 mL) was added **1** (300 mg, 1.9 mmol,1 equiv.) and 15-crown-5 (837 mg, 3.8 mmol, 2 equiv.), and the mixture was stirred for 1 h under N_2. *Cis*-1,4-dibromo-2-butene (406 mg, 1.9 mmol, 1 equiv.) in THF (10 mL) and additional 80 mL THF were then added to the reaction mixture. The reaction was allowed to stir for 1 day before it was quenched by adding several drops of water. After removal of THF on a rotavapor, the residue was purified by column chromatography (ethyl acetate/hexane = 1:1) to obtain **M1** as white solid (48 mg, yield: 12%).

^{1}H NMR (500 MHz, $CDCl_3$, ppm) δ 5.70 (t, *J* = 3.3 Hz, 2H), 4.47 – 4.30 (m, 2H), 4.11 – 3.96 (m, 2H), 3.95 – 3.77 (m, 4H), 3.59 – 3.50 (m, 2H), 3.49 – 3.40 (m, 2H), 2.44 – 2.34 (m, 2H), 2.35 – 2.23 (m, 2H).

^{13}C NMR (125 MHz, $CDCl_3$, ppm): δ 130.57, 73.49, 69.70, 67.01, 40.11, 39.15.

MS (ESI) m/z: calcd for $C_{12}H_{18}O_3Na$ [M+Na]$^+$, 233.1148; found 233.1074.

Synthesis of Biscyclooctene Ester Crosslinker

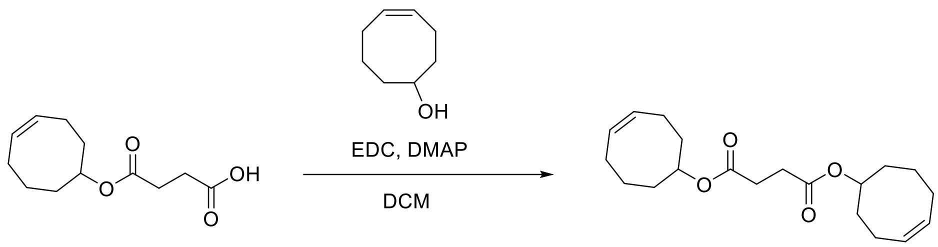

Di((Z)-cyclooct-4-en-1-yl) succinate (biscyclooctene ester crosslinker)

(Z)-4-(cyclooct-4-en-1-yloxy)-4-oxobutanoic acid[3] (1142 mg, 5 mmol,1 equiv.), 5-hydroxycyclooctene[3] (637 mg, 5 mmol, 1 equiv.), 1-ethyl-3-(3-dimethylaminopropyl)carbodiimide (EDC, 1937 mg, 10.1 mmol, 2 equiv.), and 4-dimethylaminopyridine (DMAP, 62 mg, 0.5 mmol, 0.1 equiv.) were dissolved in DCM (5 mL), and the reaction was allowed to proceed overnight. The reaction mixture was diluted with 40 mL of DCM and washed with water for three times, and the organic portion was collected and dried using sodium sulfate. After removal of DCM on a rotavapor, the residue was purified using column chromatography (diethyl ether / hexane = 1:3) to obtain the bis-cyclooctene crosslinker as colorless oil (1070 mg; yield: 64%).

^{1}H NMR (500 MHz, $CDCl_3$, ppm) δ 5.74 – 5.52 (m, 4H), 4.91 – 4.74 (m, 2H), 2.54 (d, J = 1.2 Hz, 4H), 2.38 – 2.24 (m, 2H), 2.23 – 2.12 (m, 4H), 2.12 – 2.01 (m, 2H), 1.93 – 1.77 (m, 4H), 1.70 (m, 2H), 1.65 – 1.49 (m, 6H).

^{13}C NMR (125 MHz, $CDCl_3$, ppm): δ 171.67, 129.86, 129.70, 76.07, 33.81, 33.63, 29.73, 25.66, 24.89, 22.43.

MS (ESI) m/z: calcd for $C_{20}H_{30}O_4Na$ [M+Na]$^+$, 357.2042; found 357.2026.

III. Polymer Synthesis

Synthesis of P1

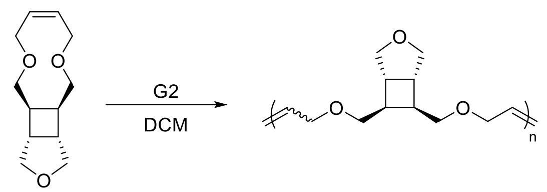

M1 **P1**

P1 of different molecular weights were made by ROMP of **M1**. The following is an example of the procedure.

To a vial was added **M1** (100 mg, 0.48 mmol, 1 equiv.) and G2 (0.4 mg, 0.000476 mmol, 0.001 equiv., in 480 μL of DCM stock solution). The reaction was gently stirred for 3 hours, and excess amount of ethyl vinyl ether (150 μL) was added and stirred for 30 minutes to terminate the polymerization. The solvent was then removed on a rotavap and subjected to preparative GPC for further purification to yield transparent viscous oil, which was dried under vacuum overnight to afford 75 mg of polymer **P1**. (75 mg, yield: 75%, M_n = 154 kDa, $Đ$ = 1.97)

^{1}H NMR (500 MHz, $CDCl_3$, ppm): δ 5.8-5.65 (m, 2H), 4.05-3.89 (m, 4H), 3.86 (d, J = 9.4 Hz, 2H), 3.65 – 3.54 (m, 2H), 3.54 – 3.41 (m, 4H), 2.64 (m, 2H), 2.35 (m, 2H).

^{13}C NMR (125 MHz, $CDCl_3$, ppm): δ 129.43, 129.38, 73.77, 71.00, 70.73, 66.81, 40.50, 38.56.

Supplementary Table 1. M_n and $Đ$ of **P1** synthesized.

[M1]/[G2]	[M1]	M_n (kDa)	$Đ$	Use
25	0.5 M	5	1.96	Sonication Control Study
400	1 M	35	1.88	Initial Sonication Study
400	1 M	58	1.65	Sonication Kinetic Study
500	1 M	68	2.12	Thermal Analysis
1000	1 M	89	1.83	Sonication Kinetic Study
1000	1 M	154	1.97	Sonication for Depolymerization Study

Synthesis of P2

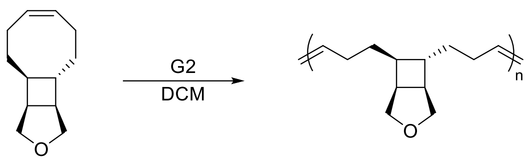

M2 P2

M2 was prepared according to previously described procedure.[1] To a vial was added **M2** (325 mg, 1.82 mmol, 1.000 equiv.) and G2 (1.55 mg, 1.82 μmol, 0.001 equiv., in 910 μL of DCM solution). The reaction was gently stirred overnight, and 100 μL ethyl vinyl ether was added and stirred for 30 minutes to quench the polymerization. The concentrated mixture was precipitated three times in cold MeOH. The collected polymer was dried under vacuum overnight to afford polymer **P2** as white fibers. (205 mg, yield: 63%, M_n = 87.5 kDa, $Đ$ = 1.83)

^{1}H NMR (500 MHz, $CDCl_3$, ppm): δ 5.43 – 5.26 (m, 2H), 4.01 (d, J = 9.8 Hz, 1H), 3.68 (d, J = 8.9 Hz, 1H), 3.42 – 3.32 (m, 2H), 2.83 – 2.73 (m, 1H), 2.48 – 2.37 (m, 1H), 2.04 – 1.75 (m, 5H), 1.53 – 1.48 (m, 2H), 1.48 – 1.29 (m, 3H).

Synthesis of PDHF (from DHF)

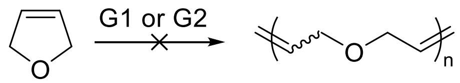

To a vial was added freshly distilled DHF (1 equiv.) and Grubbs catalyst (0.001 equiv.), and the reaction was allowed to proceed for 24 h. Excess amount of ethyl vinyl ether was added, and the mixture was stirred for 30 min before aliquots were taken for ^{1}H NMR characterization.

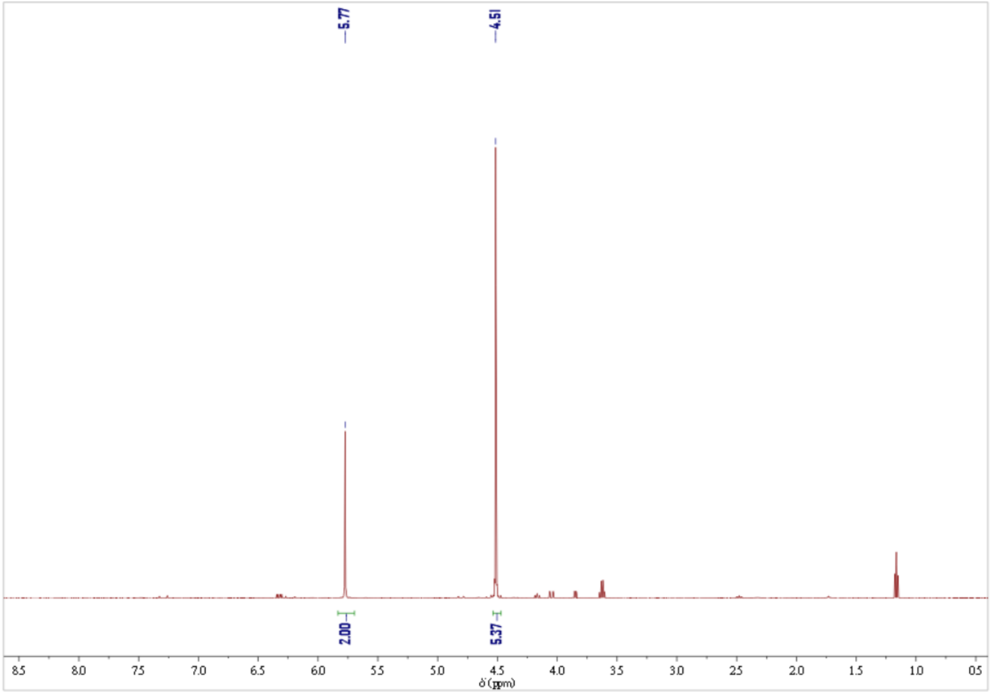

Supplementary Fig. 1: ^{1}HNMR of DHF reacting with G1.

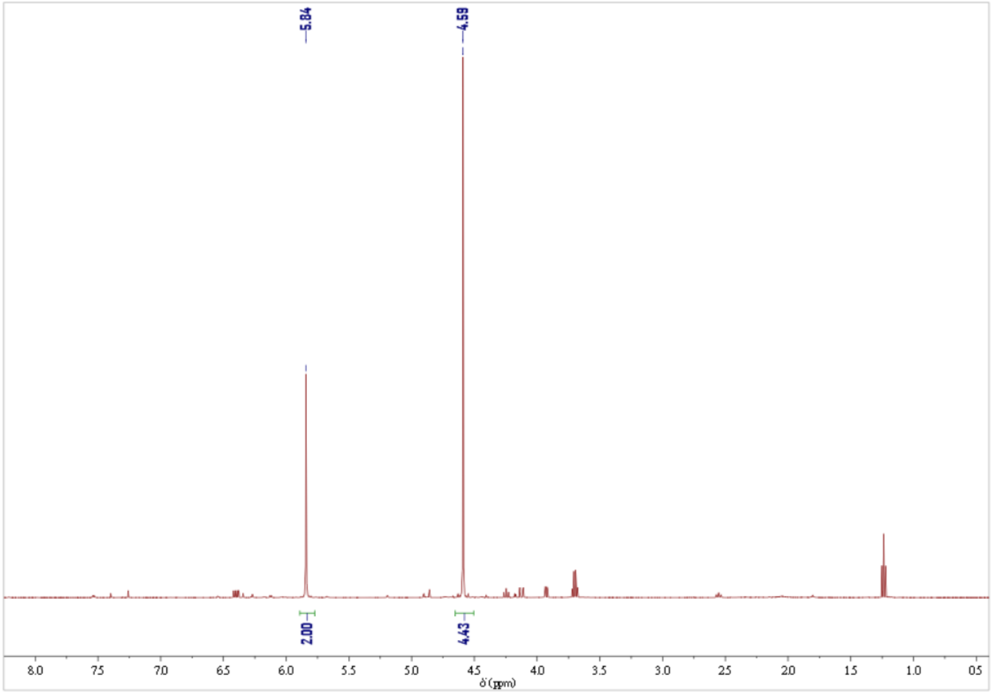

Supplementary Fig. 2: ^{1}HNMR of DHF reacting with G2.

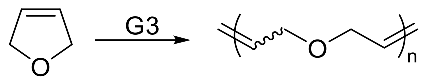

To a vial was added freshly distilled DHF (200 mg, 2.85 mmol, 1 equiv.) and G3 (3.1 mg, 0.0035 mmol, 0.00123 equiv.), and the reaction was allowed to proceed for 24 h. Excess amount of ethyl vinyl ether (200 µL) was added, and the mixture was stirred for 30 min. The reaction mixture was precipitated in cold methanol to obtain PDHF as a brown oil (17 mg, yield: 9%, M_n = 14 kDa, $Đ$ = 2.12).

^{1}H NMR (500 MHz, $CDCl_3$, ppm): δ 5.87 – 5.67 (m, 2H), 4.10 – 3.84 (m, 4H).

Synthesis of PDHF (from DHF dimer)

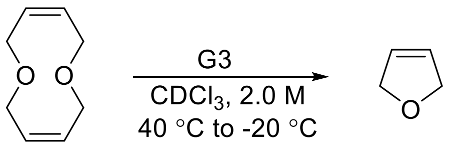

DHF dimer

DHF dimer (60 mg, 0.43 mmol, 1 equiv.) and a stir bar was charged in a small vial. **G3** (0.38 mg, 0.00043 mmol, 0.001 equiv., in 143 µL of $CDCl_3$ stock solution) was added. The vial was sealed and heated to 40 °C, and the reaction was stirred at this temperature for 5 min. The vial was then put in a freezer (-20 °C) and left there for 16 h. The reaction was taken out from the freezer, and excess amount of cold ethyl vinyl ether (100 µL) was added to quench the reaction. The vial was put back to freezer left there for 30 min before ^{1}H NMR characterization.

^{1}H NMR (500 MHz, $CDCl_3$, ppm): δ 5.90 – 5.87 (m, 2H), 4.68 – 4.52 (m, 4H).

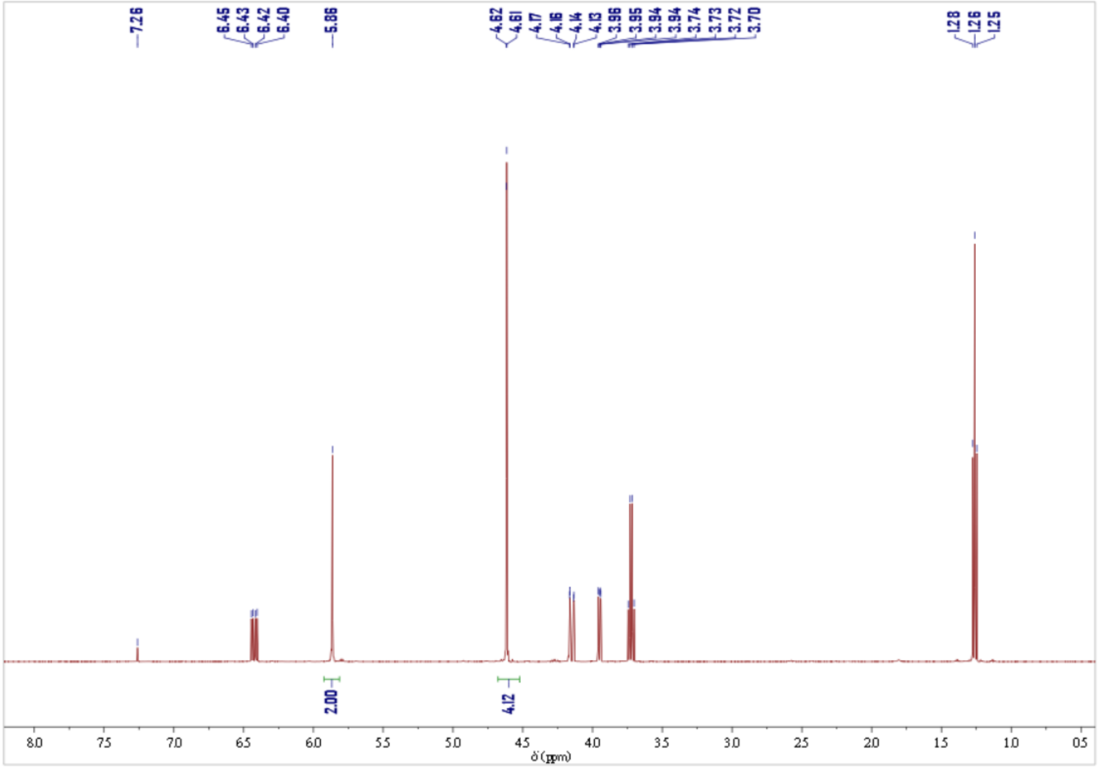

Supplementary Fig. 3: ^{1}HNMR of DHF dimer reacting with G3.

IV. Thermal Properties of Polymers and Networks

Thermogravimetric analysis (TGA) was performed using a TA Discovery TGA 550 at a ramp rate of 10 °C/min under nitrogen flow. Thermal decomposition onset temperature T_d was reported at the temperature at which 5% mass loss. Differential scanning calorimetry (DSC) was determined with a TA Instrument of Discovery DSC 250 using hermetic aluminum pans under nitrogen atmosphere using a heat-cool-heat cycle at a heating ramp rate of 10 °C/min and a cooling ramp rate of 5 °C/min. Results were processed with TA TRIOS software and shown from the cooling and the second heating curve.

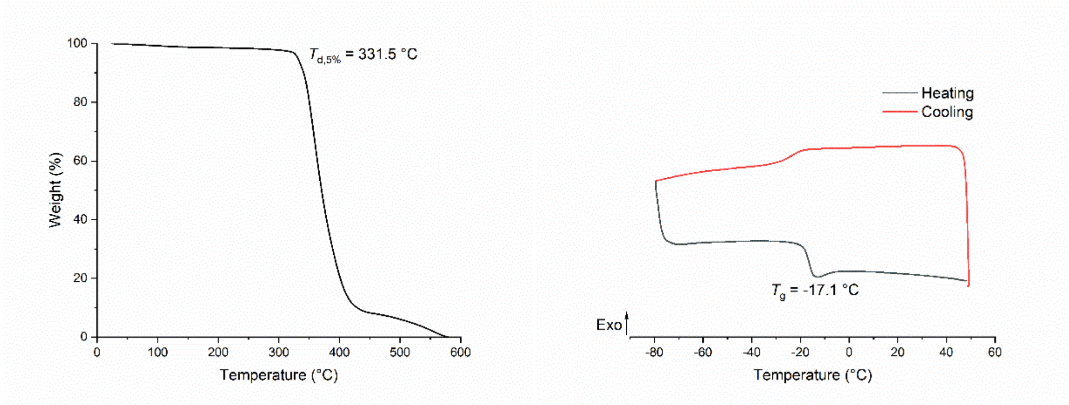

Supplementary Fig. 4: Thermal characterizations: TGA curves (left) and DSC curves (right) of **P1 (**68 kDa, *Đ* = 2.12).

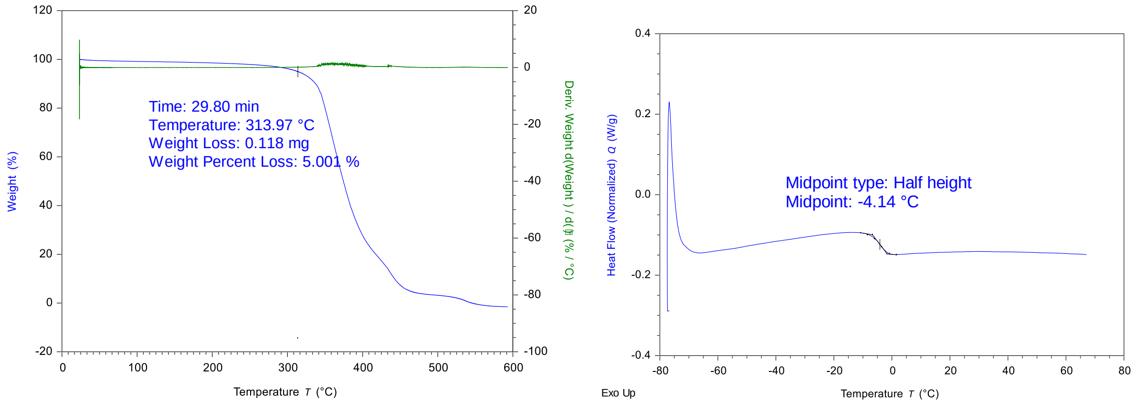

Supplementary Fig. 5: Thermal characterizations: TGA curves (left) and DSC curves (right) of **PN1**.

V. Sonication Experiments

General

Sonication experiments were conducted using a Vibracell model VCX500 (Sonics & Materials) with a standard solid probe (tip diameter = 13 mm, titanium alloy Ti-6Al-4V) operated at 20 kHz. 1 mg/1 mL THF solution of **P1** was made and transferred to a reaction vessel (made by U Akron Glassblowing Shop) and was then deoxygenated by bubbling through N_2 for 30 minutes. The solution was sonicated under N_2 using a pulse sequence 1 s on / 1 s off at an energy density of 9.3 W/cm^2 (AMPL = 25%). The energy density was calibrated according to a previously reported procedure.[4] The temperature of the solution was maintained by placing the vessel in an ice/water bath. Ring opening percentage and molecular weight changes were tracked by ^{1}H NMR and GPC, respectively.

Calculation of % Ring Opening of P1 from ^{1}H NMR

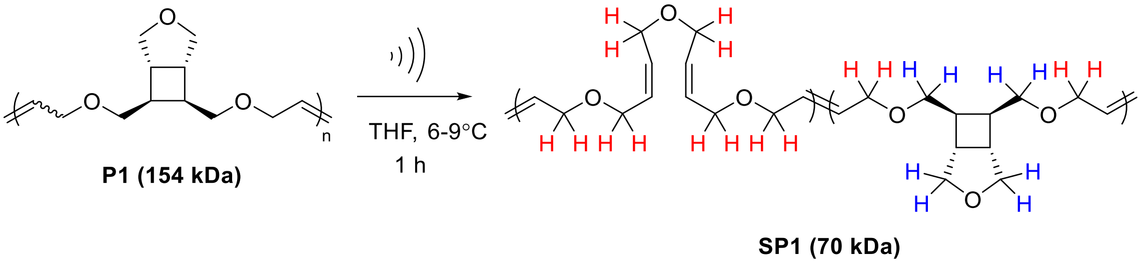

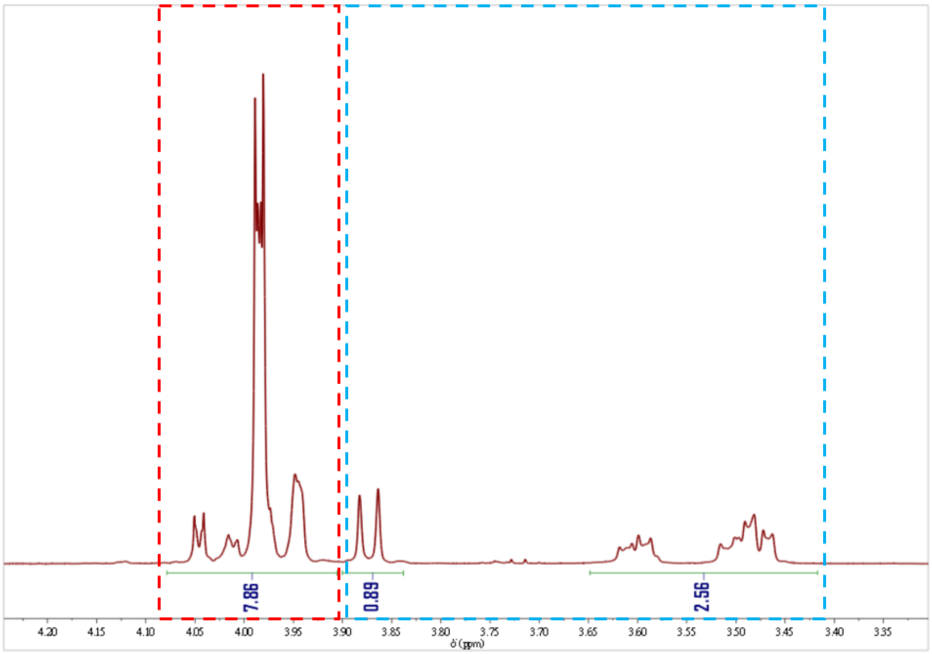

Supplementary Fig. 6: ^{1}H NMR for 1 h of sonication of 154 kDa **P1**.

$$8 \text{ methylene H (blue) in nonactivated region} = 2.56 + 0.89 = 3.45$$

$$\text{nonactivated region allylic H (red)} = \frac{3.45}{8} \times 4 = 1.725$$

$$\text{activated region allylic H (red)} = 7.86 - 1.725 = 6.135$$

$$\text{activated: nonactivated} = \frac{6.135}{12} : \frac{1.725}{4} = 0.511: 0.431$$

$$\%\text{RO} = \frac{0.511}{0.511 + 0.431} = 54\%$$

Calculation of Z/E Ratio for Mechanochemically Generated Olefins from P1 to SP2

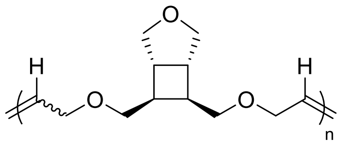

P1 (89 kDa)

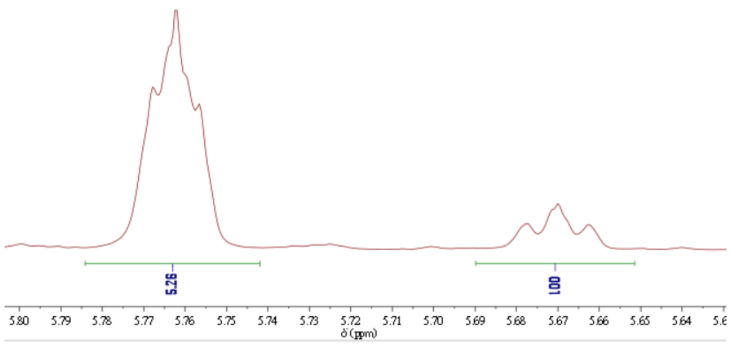

Supplementary Fig. 7: ^{1}HNMR for **P1** (olefin region).

$$\text{original olefin: E form : Z form} = \frac{5.26}{1}$$

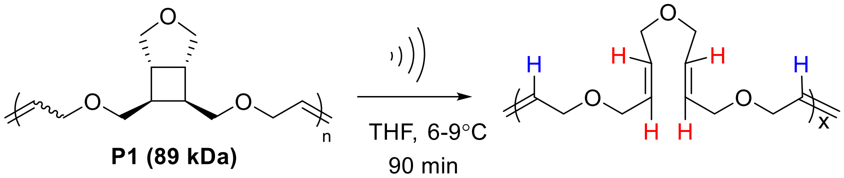

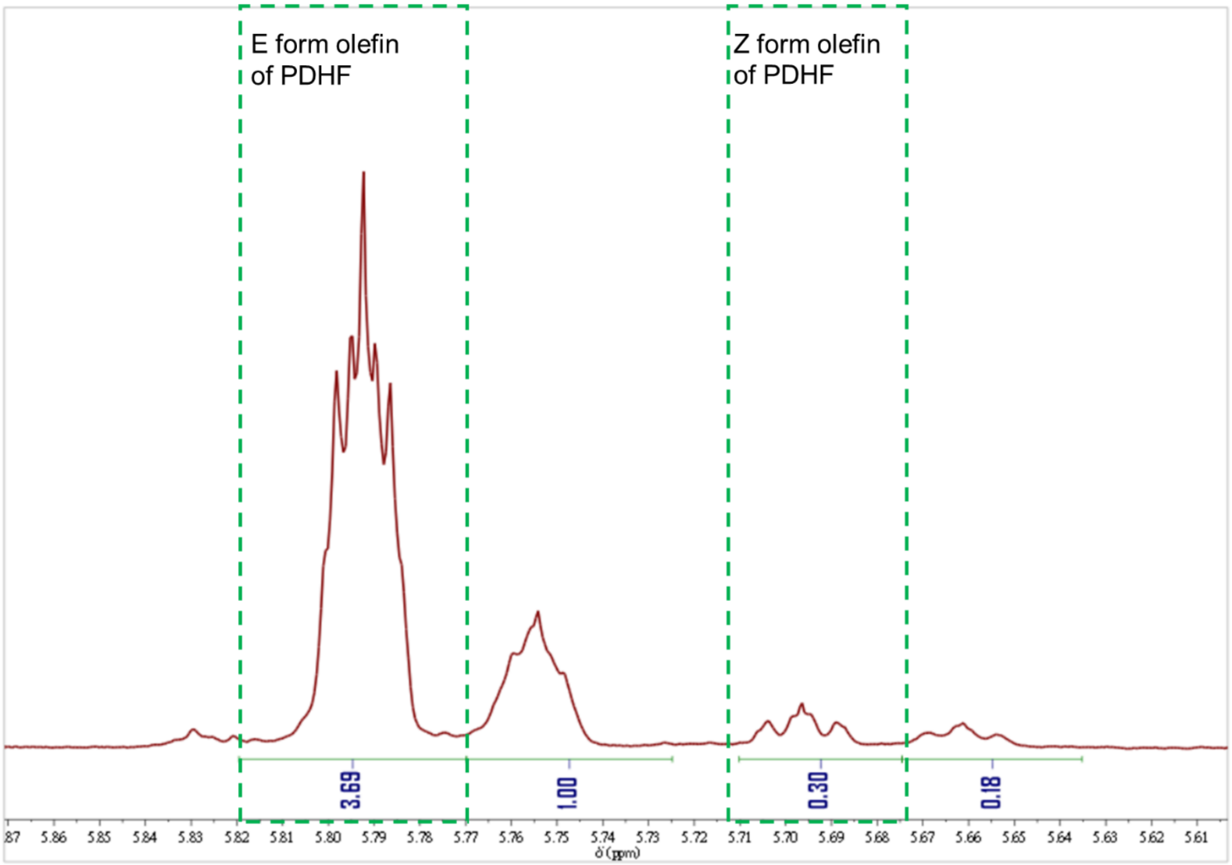

Supplementary Fig. 8: ^{1}HNMR for **SP1** (olefin region).

$$\text{PDHF olefinic H} = 3.69 + 0.3 = 3.99$$

$$\text{blue H (original olefin)} = \frac{3.99}{6} \times 2 = 1.33$$

$$\text{E form of original olefin} = \frac{5.26}{1 + 5.26} \times 1.33 = 1.12$$

$$\text{Z form of original olefin} = \frac{1}{1 + 5.26} \times 1.33 = 0.21$$

$$\text{E form of new olefin} = 3.69 - 1.12 = 2.57$$

$$\text{Z form of new olefin} = 0.3 - 0.21 = 0.09$$

$$\frac{\text{Z}}{\text{E}} \text{ ratio of new olefins} = \frac{0.09}{2.57} = 0.035$$

Calculation of % Ring Opening of P2 from ^{1}H NMR

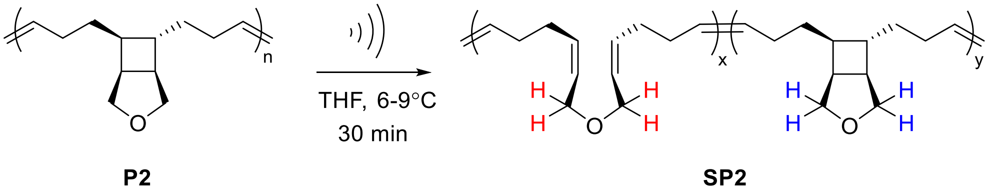

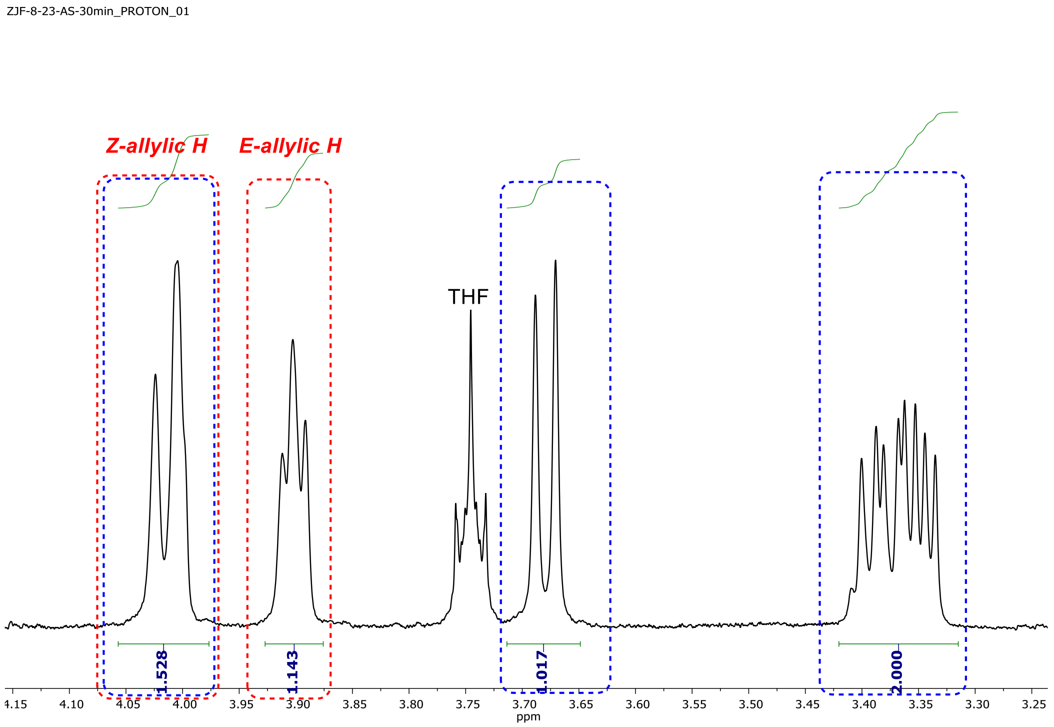

Supplementary Fig. 9: Partial ^{1}HNMR for 88 kDa **SP2** after 30 minutes of sonication.

(**Note**: two peaks overlapped in the region from 4.05 ppm to 3.98 ppm)

$$\text{THF H (blue)} = 1.017 \times 2 + 2.000 = 4.034$$

$$\text{allylic H (red)} = 1.528 - 1.017 + 1.143 = 1.654$$

$$\frac{x}{y} = \frac{1.654}{4.034}$$

$$\%\mathrm{RO} = \frac{x}{x+y} = \frac{1.654}{1.654 + 4.034} = 29.1\%$$

Calculation of Z/E Ratio for Mechanochemically Generated Olefins from P2 to SP2

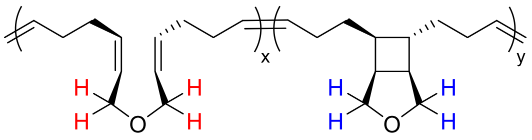

SP2

$$\frac{\mathrm{Z}}{\mathrm{E}} = \frac{1.528 - 1.017}{1.143} = 0.447$$

Supplementary Table 2. Summary of %RO and Z/E ratio after sonication for 89 kDa **P1** and 88 kDa **P2.**

SP1			SP2			
Time	%RO	Z/E	Time	%RO	Z/E	%EZ
2 min	6.5%	0.0755	2 min	4.3%	0.1030	18.7%
4 min	12.1%	0.0628	4 min	8.0%	0.3170	48.1%
8 min	20.0%	0.0329	8 min	13.4%	0.3995	57.1%
15 min	28.2%	0.0352	15 min	19.8%	0.3657	53.6%
30 min	37.6%	0.0374	30 min	29.1%	0.4470	61.8%
60 min	47.5%	0.0369				
90 min	52.7%	0.0341				

Sonication Control Study for 5 kDa P1

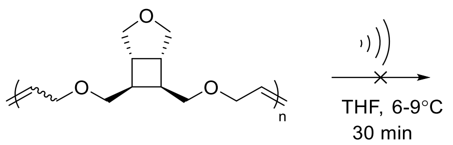

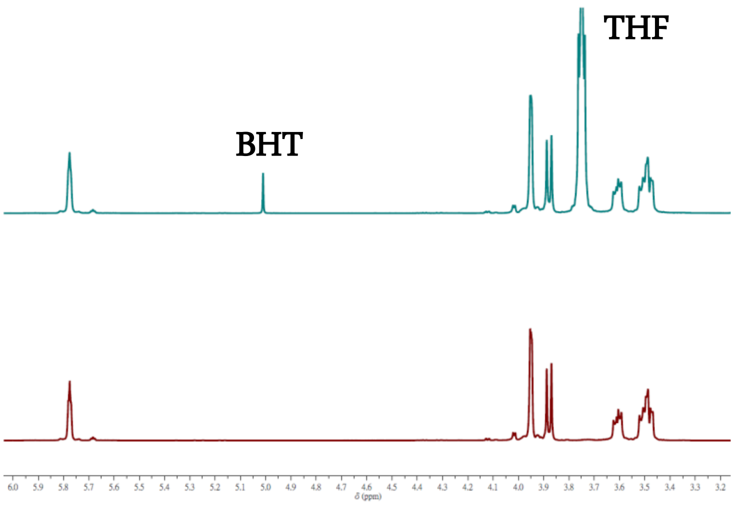

Supplementary Fig. 10: ^{1}HNMR for 5 kDa **P1** before sonication (bottom, red) and after sonication (top, blue).

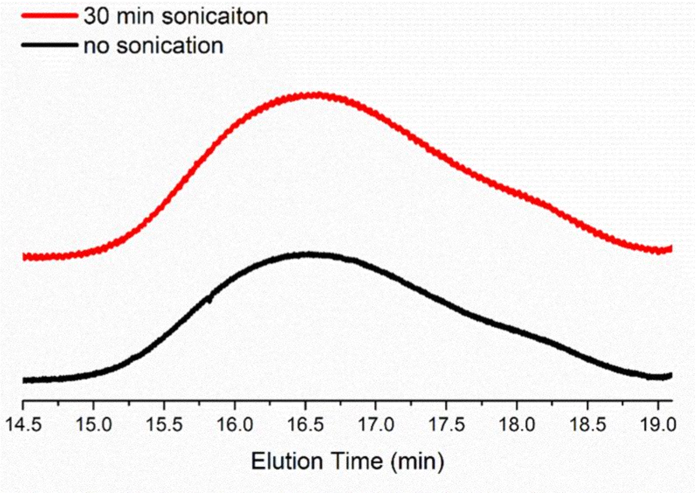

Supplementary Fig. 11: GPC for 5 kDa **P1** before sonication (bottom, black) and after sonication (top, red).

Calculation of Scission Cycle from M_n Changes[5]

$$\mathrm{SC} = [\ln(M_{n(0)}) - \ln(M_{n(t)})]/\ln(2)$$

For 89 kDa **P1** sonicated 90 minutes:

$$\mathrm{SC} = [\ln(89375) - \ln(41546)]/\ln(2) = 1.11$$

Extent of Ring Opening (ϕ) vs Scission Cycle

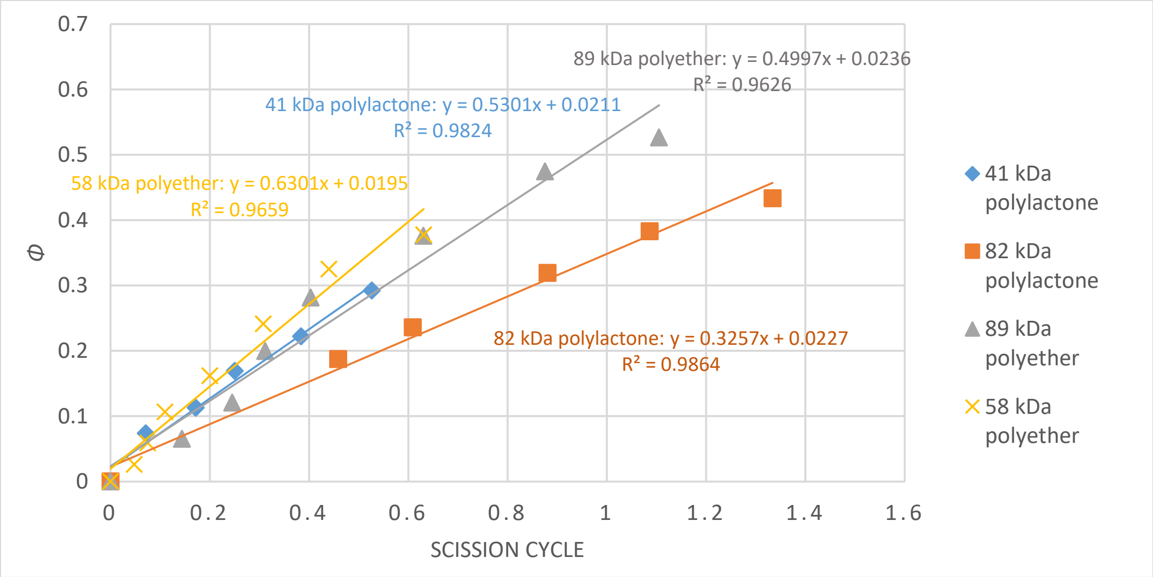

Supplementary Fig. 12: Extent of ring opening (ϕ) vs scission cycle for **P1** (this work) and a poly(*trans*-cyclobutane-fused lactone) of different initial molecular weights (reported previously[6]). The extent of ring opening per chain scission ϕ = 0.63 and 0.50 for 58 kDa and 89 kDa **P1**, respectively; ϕ = 0.53 and 0.33 for 41 kDa and 82 kDa poly(*trans*-cyclobutane-fused lactone), respectively.

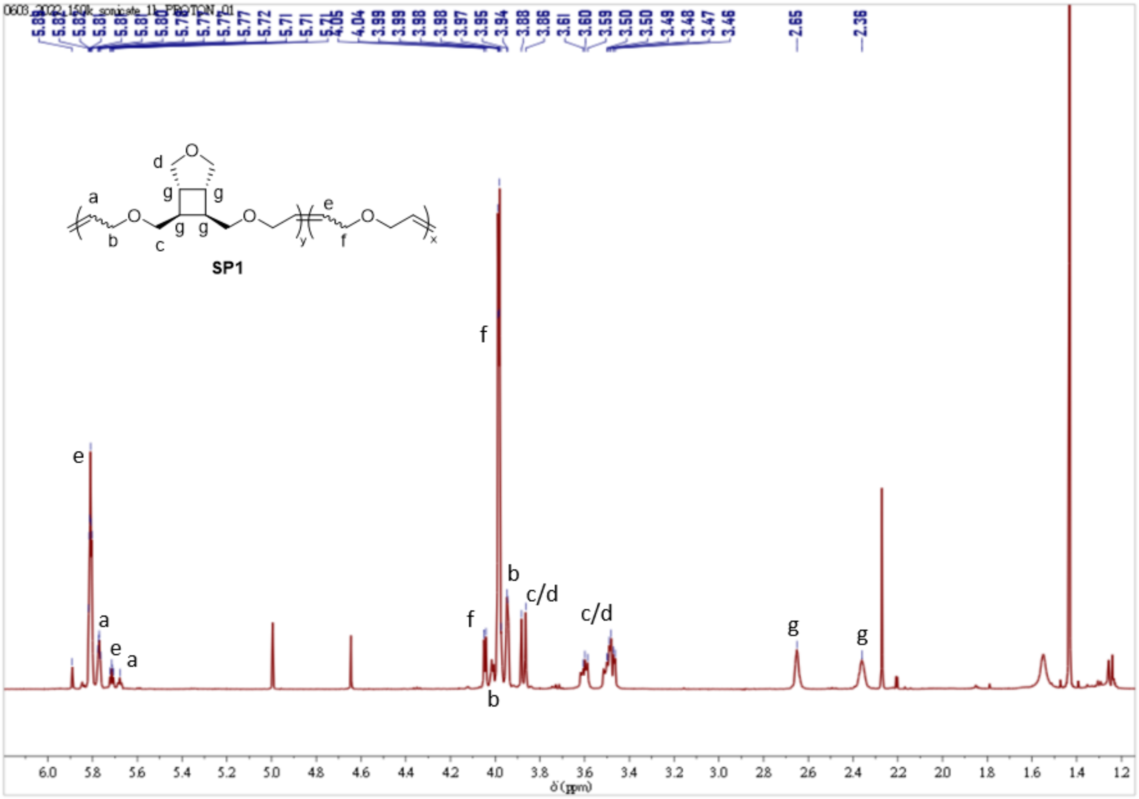

Supplementary Fig. 13: ^{1}H NMR for **SP1**.

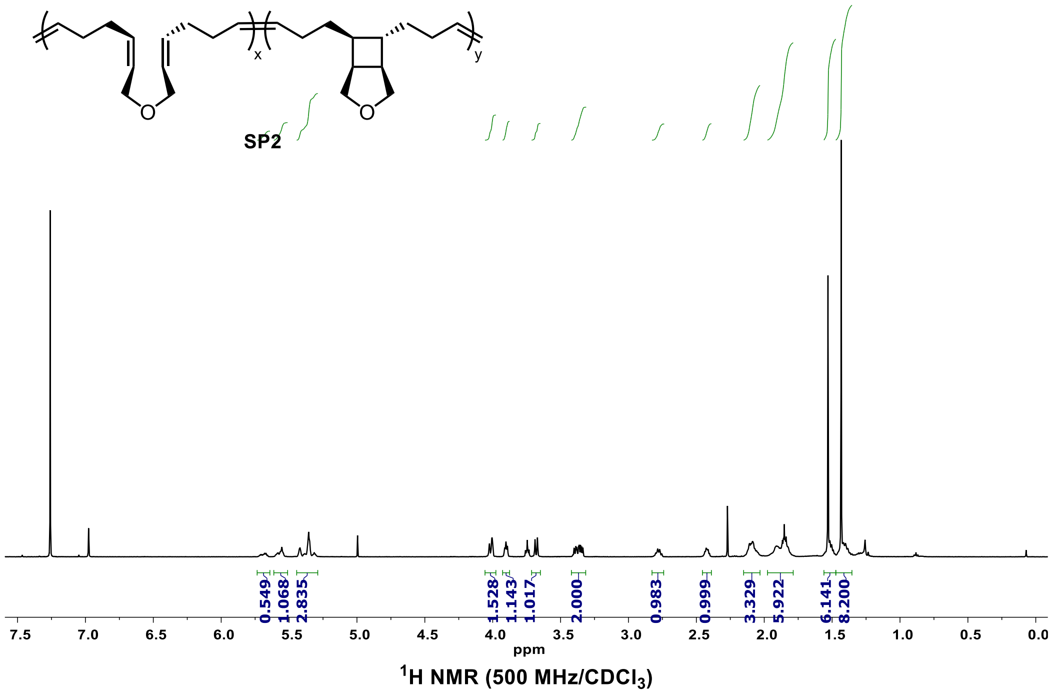

Supplementary Fig. 14: ^{1}H NMR for **SP2**.

VI. Depolymerization Experiments

General

The sonicated sample **SP1** (33 mg, %RO = 68%) was dried and reacted with 0.8 mol% of G2 (2.7 mg, 0.0031 mmol, 0.008 equiv., in 6.3 mL of degassed $CDCl_3$ stock solution) to olefin (0.06 M, 1.0 equiv) under the protection of N_2 for 6 hours at 30 °C. Aliquots were taken out at different reaction periods, and excess amount of ethyl vinyl ether (100 μL) was added and left standing for 30 minutes before ^{1}HNMR and GPC analysis.

Calculation of Depolymerization Percentage

6 hours of depolymerization of 44 kDa **SP1** with 68% ring opening is used as an example for the calculation:

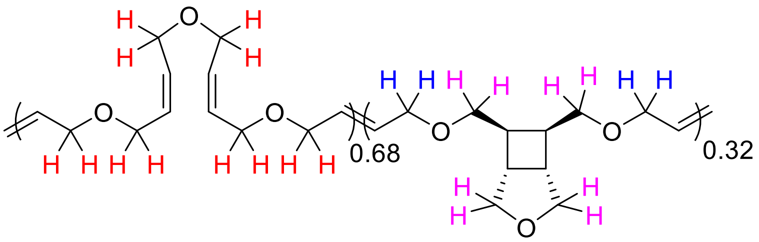

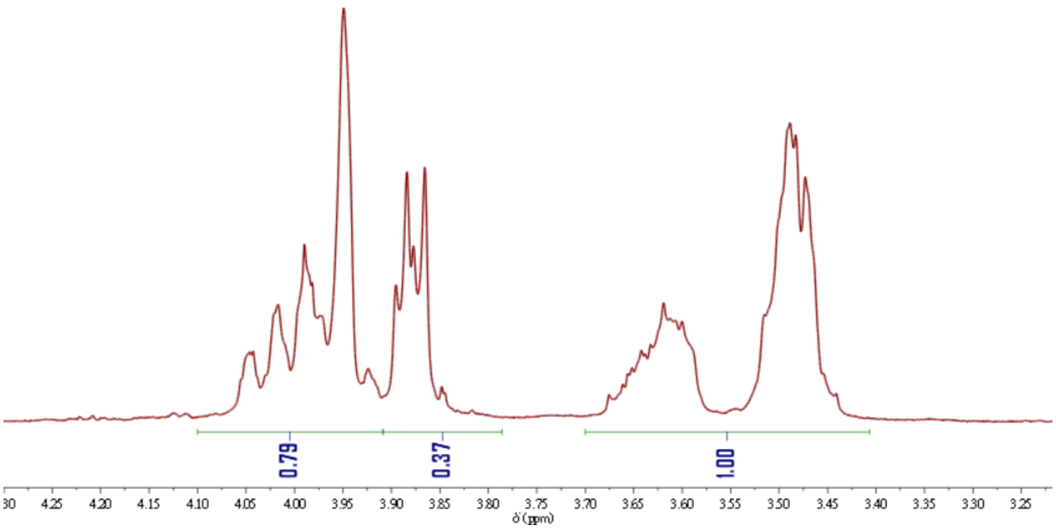

Supplementary Fig. 15: ^{1}HNMR of **SP1** reacting with G2 for 6h.

$$\text{aliphatic H (pink H)} = 1 + 0.37 = 1.37$$

$$\text{inactivated } \mathbf{P1} \text{ H (blue H)} = \frac{1.37}{8}(4) = 0.685$$

$$\frac{0.685}{4} = 0.32n \Rightarrow n = 0.535$$

$$\text{activated allylic H (red H)} = 0.79 - 0.685 = 0.105$$

$$\text{activated allylic H (red H)} = \frac{0.105}{12} = 0.00875 = 0.01635n$$

$$= \text{nondepolymerized activated region}$$

$$\text{depolymerization fraction} = \frac{0.68\text{n} - 0.01635n}{0.68n}$$

$$= \frac{(\text{activated region}) - (\text{nondepolymerized activated region})}{\text{activated region}}$$

$$= 0.975 \sim 0.98$$

Depolymerization Results for 44 kDa SP1

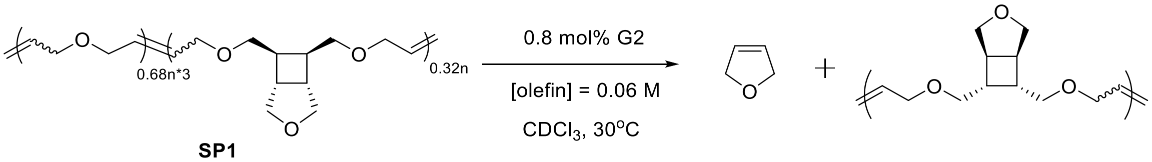

SP1

Supplementary Table 3. Summary of depolymerization kinetic study for 44kDa **SP1.**

Reaction Time (min)	Depolymerization (%)	M_n (Da)
360	97.6	4889
240	97.4	5159
120	95.6	6344
60	88.4	8376
30	69.6	13389
15	46.0	18436
10	42.3	20527
5	36.3	21170
0	0	44238

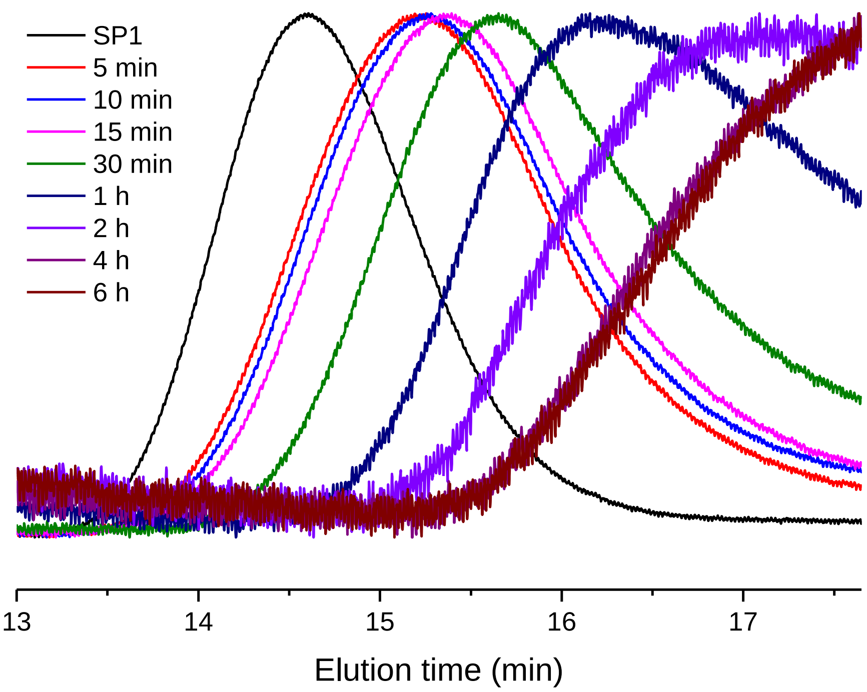

Supplementary Fig. 16: GPC traces of depolymerized **SP1** with different reaction time.

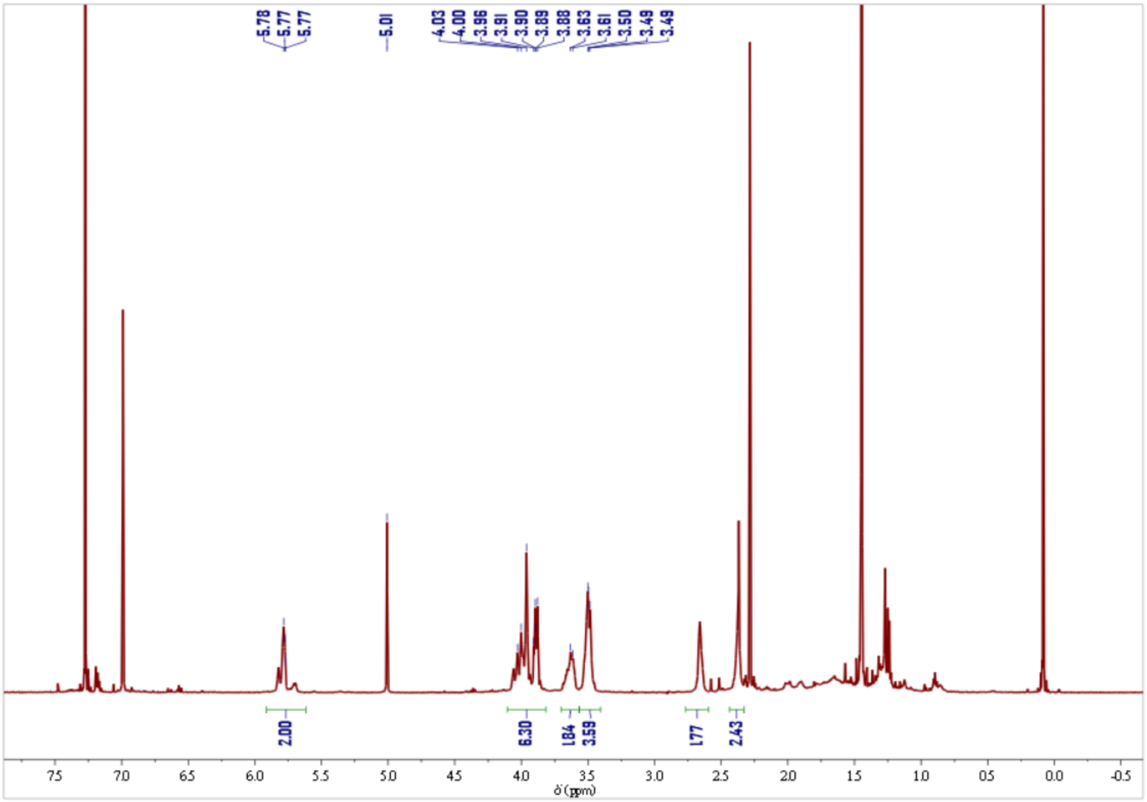

Supplementary Fig. 17: ^{1}HNMR of **SP1** reacting with G2.

Mass Spectrometry and ^{1}HNMR Results for P1 Reacting with G2

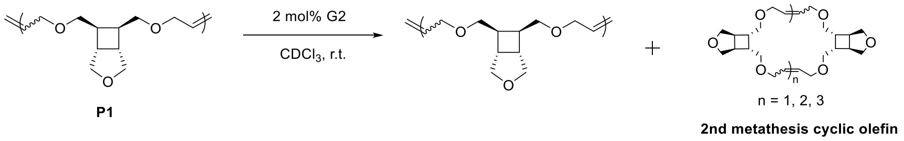

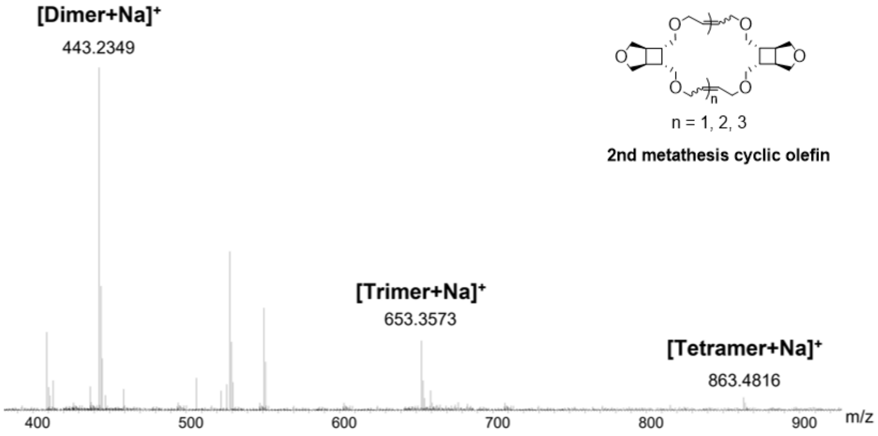

Supplementary Fig. 18: Mass spectrometry of **P1** reacting with G2 for 1 day.

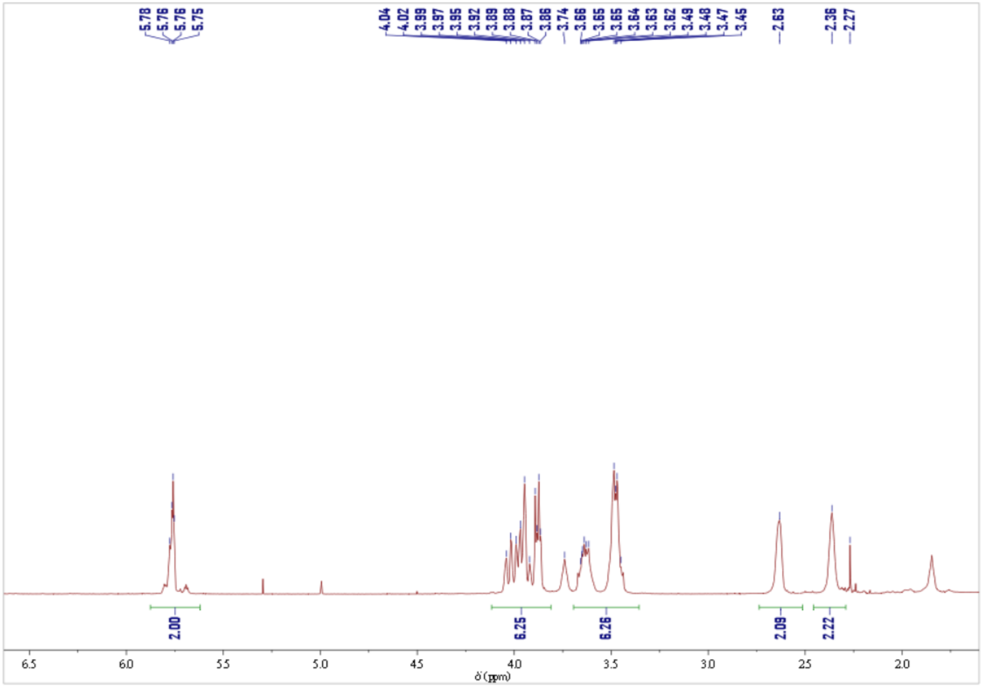

Supplementary Fig. 19: ^{1}HNMR of **P1** reacting with G2 for 1 day.

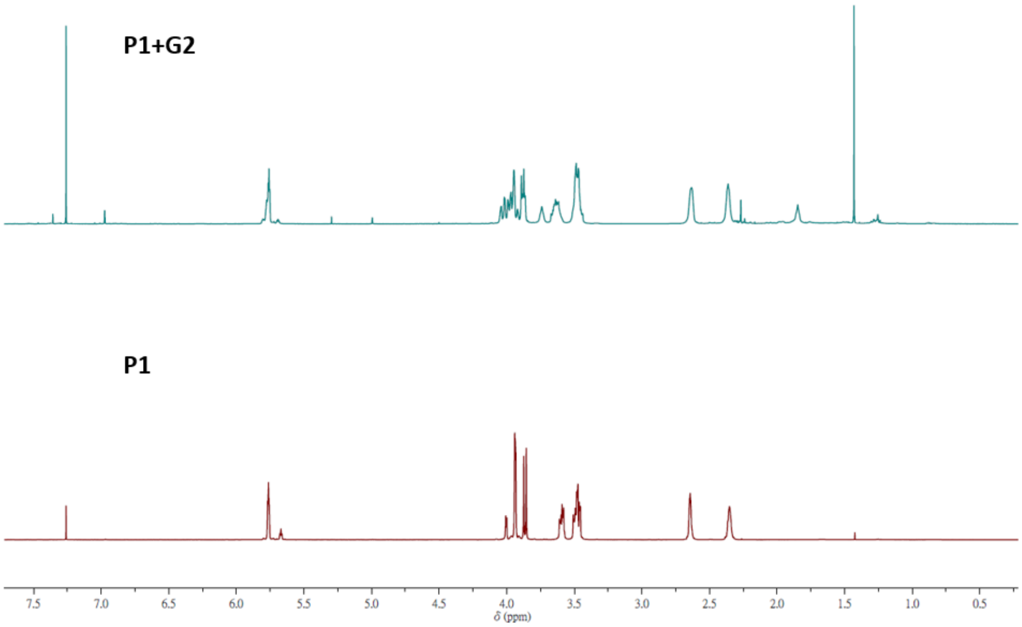

Supplementary Fig. 20: Comparison of ^{1}HNMR of **P1** (bottom, maroon) and **P1** reacting with G2 for 1 day (top, blue).

VII. Bulk Activation

Extrusion

P1 (200 mg, M_n = 69 kDa, $Đ$ = 1.80) in DCM (10 mL) was added to a solution of polyethylene glycol 2000 (20 g) in DCM (50 mL), and the mixture was dried and ground using a mortar and pestle to afford white flakes. The polymer blend was then subjected to extrusion on a twin screw extruder (Prism USALAB 16, Thermo Electron Co.,UK). The barrel temperature was set at 70 °C, and the flow rate was regulated by varying the material feeding rate. The extruded product was dissolved in a mixture of 5% DCM/water, and the solution was stirred overnight. The organic portion was collected, concentrated, and added dropwise to deionized water, and the resulting mixture was stirred overnight. The floated portion was collected and characterized by ^{1}H NMR and GPC (M_n = 6 kDa, $Đ$ = 4.89). Subsequent depolymerization study was carried out by mixing extruded **P1** (7 mg) with $CDCl_3$ solution of G2 (0.6 mg, 0.0007 mmol, 0.02 equiv). After being stirred at 30 °C overnight, the reaction was quenched by ethyl vinyl ether (50 μL) and characterized by ^{1}H NMR and GPC.

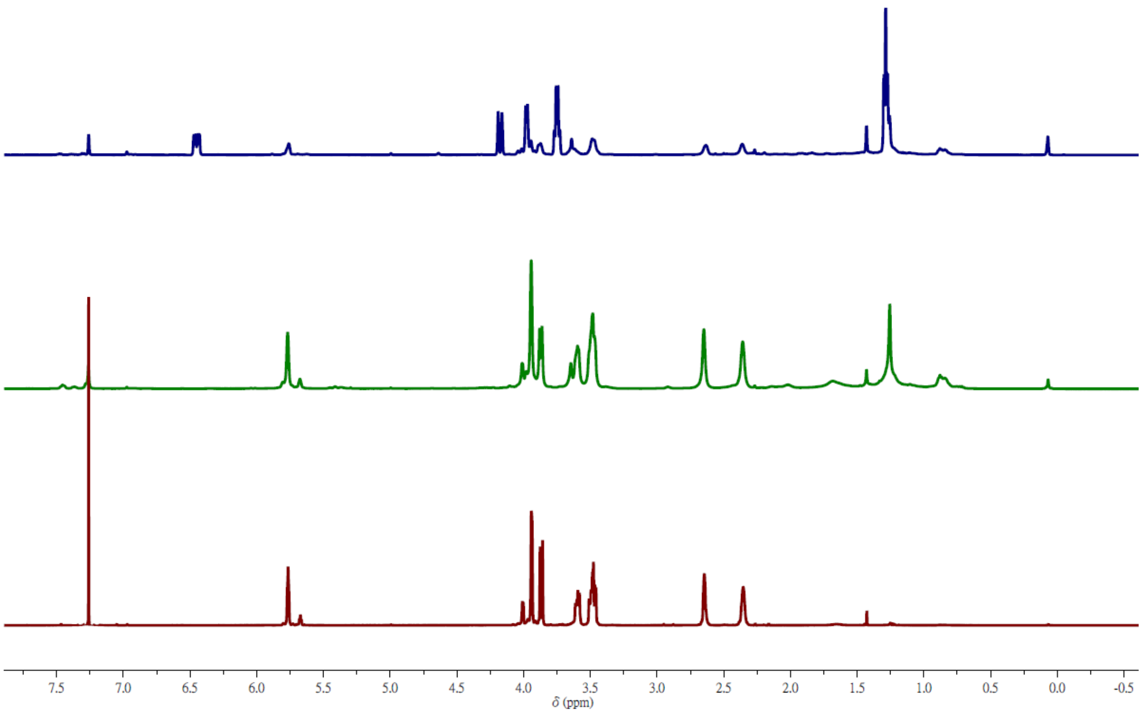

Supplementary Fig. 21: ^{1}HNMR for extruded **P1** reacting with G2 overnight (top,

blue), **extruded P1** (middle, green), and **P1** (bottom, maroon).

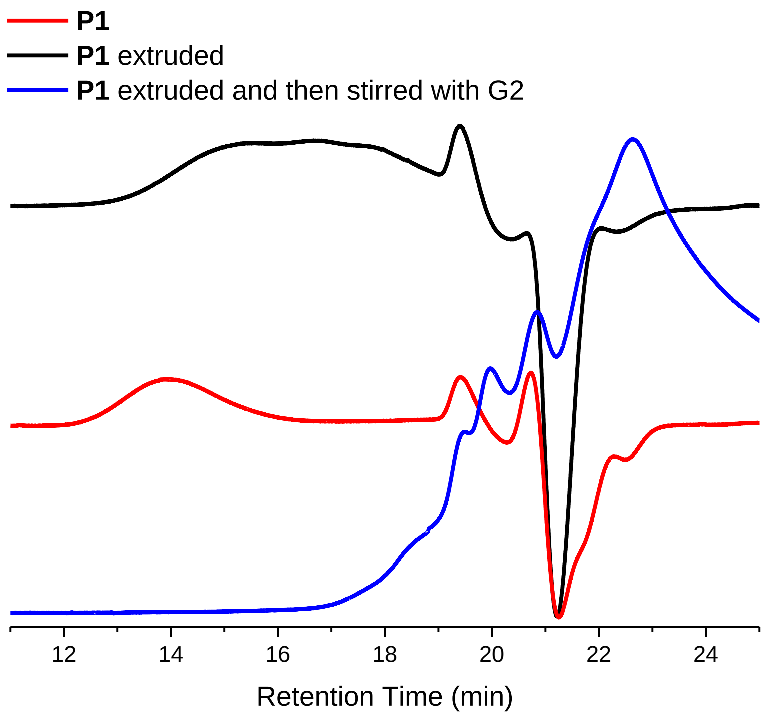

Supplementary Fig. 22: GPC traces of **P1** (middle, red), extruded **P1** (top, black)**,** and extruded **P1** stirred with G2 (bottom, blue).

Polymer Network PN1 Synthesis

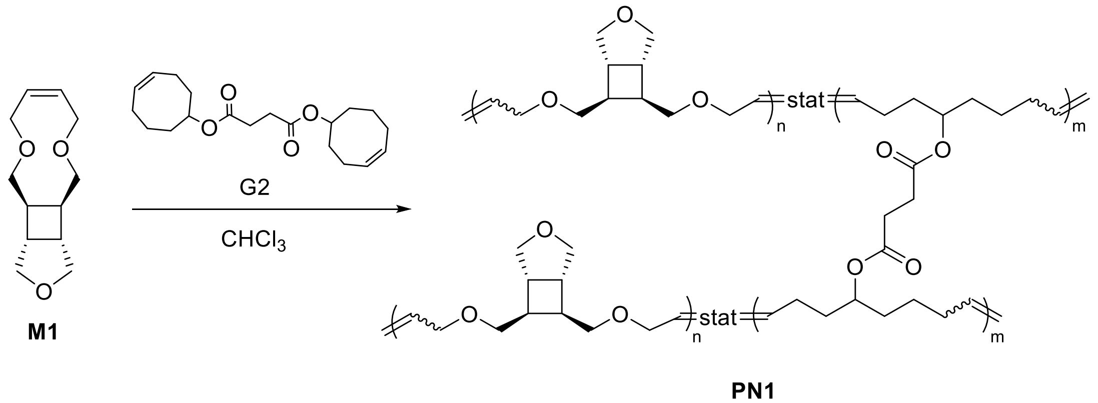

M1 (273 mg, 1.3 mmol, 1 equiv.) and bis-cyclooctene crosslinker (48 mg, 0.14

mmol, 0.1 equiv.) were dissolved in chloroform (200 μL) in a beaker, and to the solution was added G2 (2.7 mg, 0.003 mmol, 0.0025 equiv). The reaction was allowed to proceed overnight, resulting in a thin film with brown color, which was carefully peeled off and soaked in 1/1 v/v ethyl vinyl ether/chloroform for a day. The film was further washed through Soxhlet extraction using THF as the solvent to obtain a transparent film **PN1**.

Compressive Testing of PN1

Three samples of **PN1** with sample area of 4 mm × 4 mm and thickness of 0.75 mm were subjected to compression using Instron 5969. Compression strain rate was set to 5 mm/mm/min. Water was used as the lubricant to avoid shear and friction between samples and steel plate. Young's modulus is 1.03 ± 0.19 MPa based on measurement between 5-10% strain.

Ball Milling

In the grinding jar of Retsch CryoMill was charged **PN1** and a steel ball (diameter: 2.5 cm). The grinding was allowed to proceed for 10 minutes at a frequency of 25 Hz at room temperature. Viscous white solids were collected for further experiments.

Depolymerization Study for Bulk Activation Sample

P1 or **PN1** was dissolved or swollen in $CDCl_3$ with [olefin] = 0.06 M, and 2 mol% G2 was added. The mixture was allowed to stir overnight at 30 °C until the reaction was quenched by excess amount of ethyl vinyl ether. For **PN1**, BHT was added as the internal standard to analyze the percentage of DHF generation.

Calculation of % DHF in Depolymerization of Ball-Milled PN1

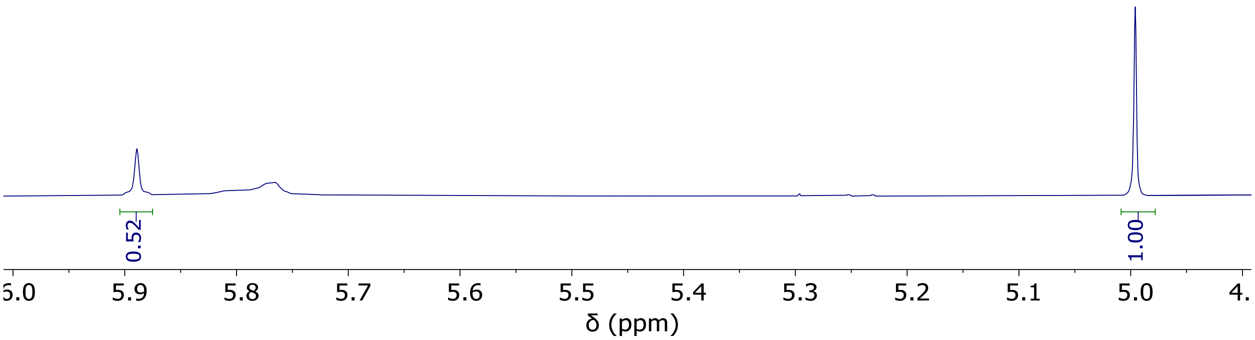

Supplementary Fig. 23: Partial ^{1}HNMR of **PN1** after ball milling and then treated with G2 (BHT as internal standard).

$$2.6\, mg\ BHT \times \frac{1\, mol\ BHT}{220.35\ g\ BHT} \times \frac{0.26\, mol\ DHF}{1\ mol\ BHT} = 3.07\ \mu mol\ DHF$$

$$12\ mg\ \boldsymbol{PN1} \times \frac{1\ mol\ repeat\ unit}{222.4\ g\ repeat\ unit} \times \frac{0.9\ mol\ \boldsymbol{M1}}{1\ mol\ repeat\ unit} = 48.6\ \mu mol\ \boldsymbol{M1}$$

$$\therefore The\ amount\ of\ DHF\ retrieved\ was\ 6\ mol\%\ to\ the\ amount\ of\ \boldsymbol{M1}$$

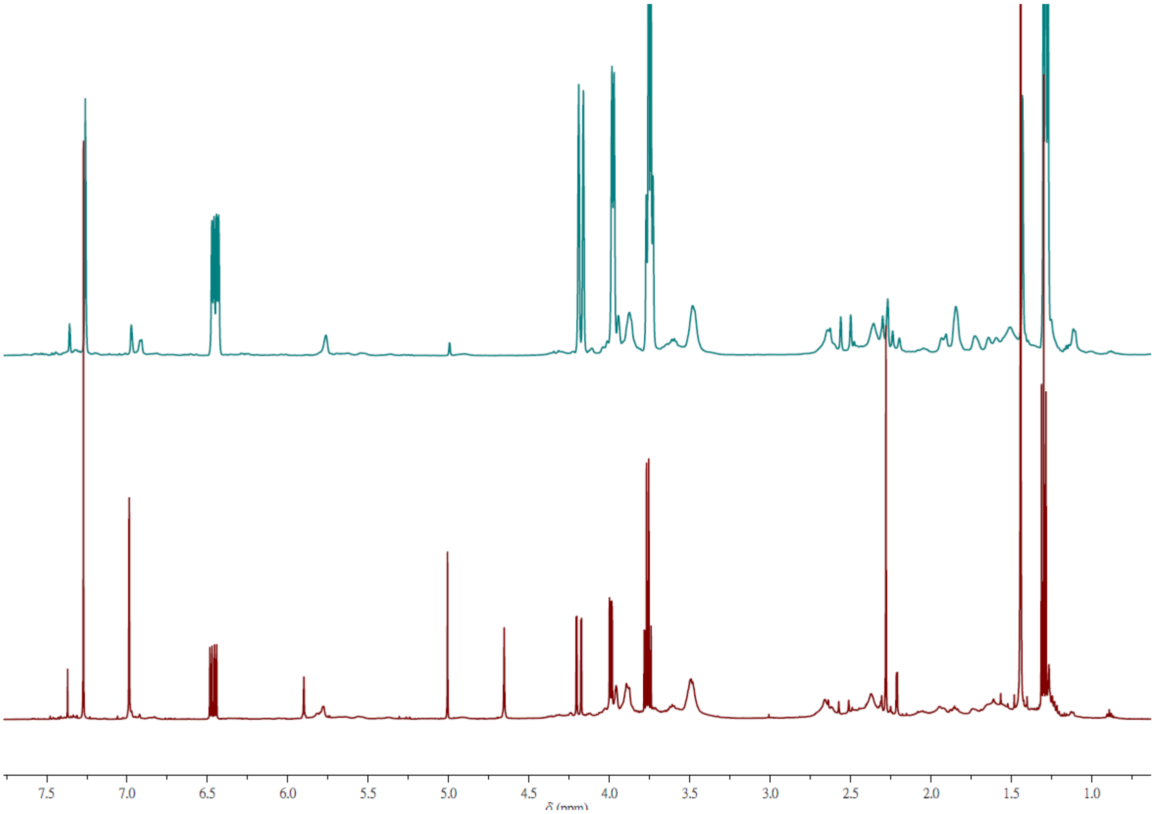

Supplementary Fig. 24: ^{1}HNMR for **PN1** compressed (top, blue) and ball milled (bottom, maroon) after treating with G2 overnight.

Degradation Study for Ball Milled Sample

Ball milled **PN1** (16 mg) was added to a solution of 0.1 M KOH (5/95 H_2O/DMSO), and the mixture was stirred overnight. To the mixture was added chloroform (10 mL), and the organic portion was collected, washed with deionized water (10 mL × 3), and dried by sodium sulfate. Organic solvent was removed on a rotavap, and the sample was further purified using a preparative GPC, affording a colorless viscous oil (4 mg).

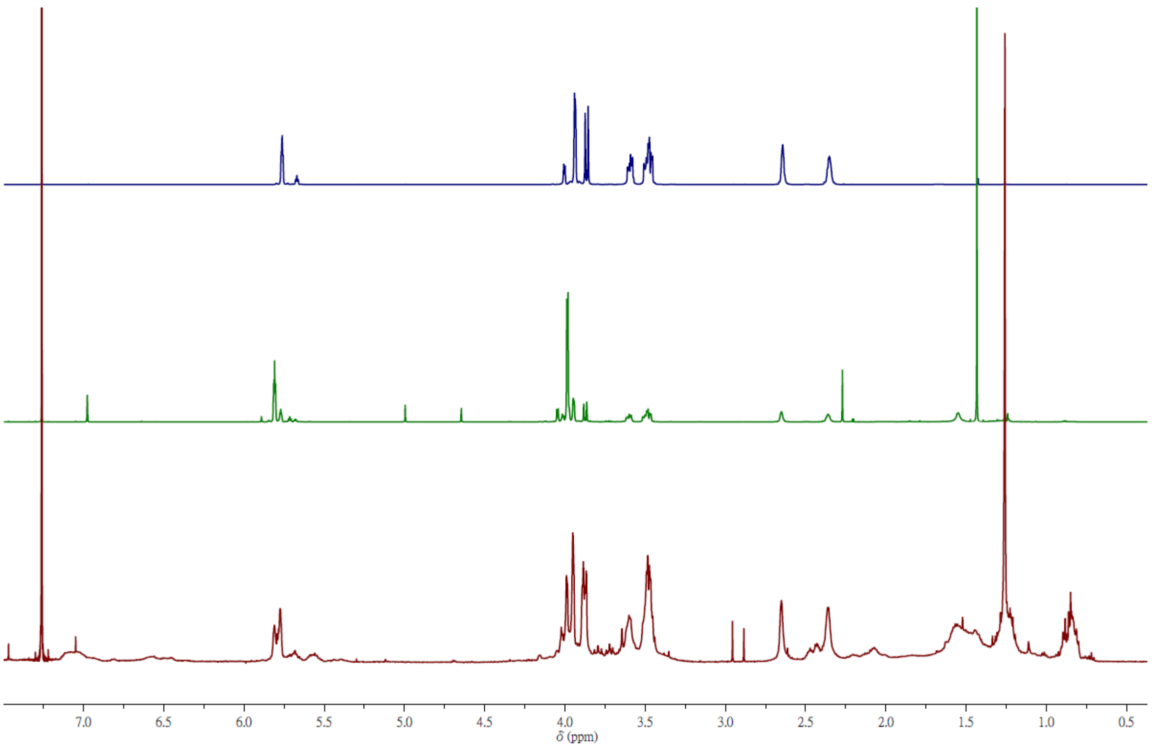

Supplementary Fig. 25: Comparison of ^{1}HNMR of **P1** (top, blue), **SP1**. (middle, green), and ball-milled **PN1** degradation product (bottom, red)

Calculation of % Ring Opening in Degraded PN1

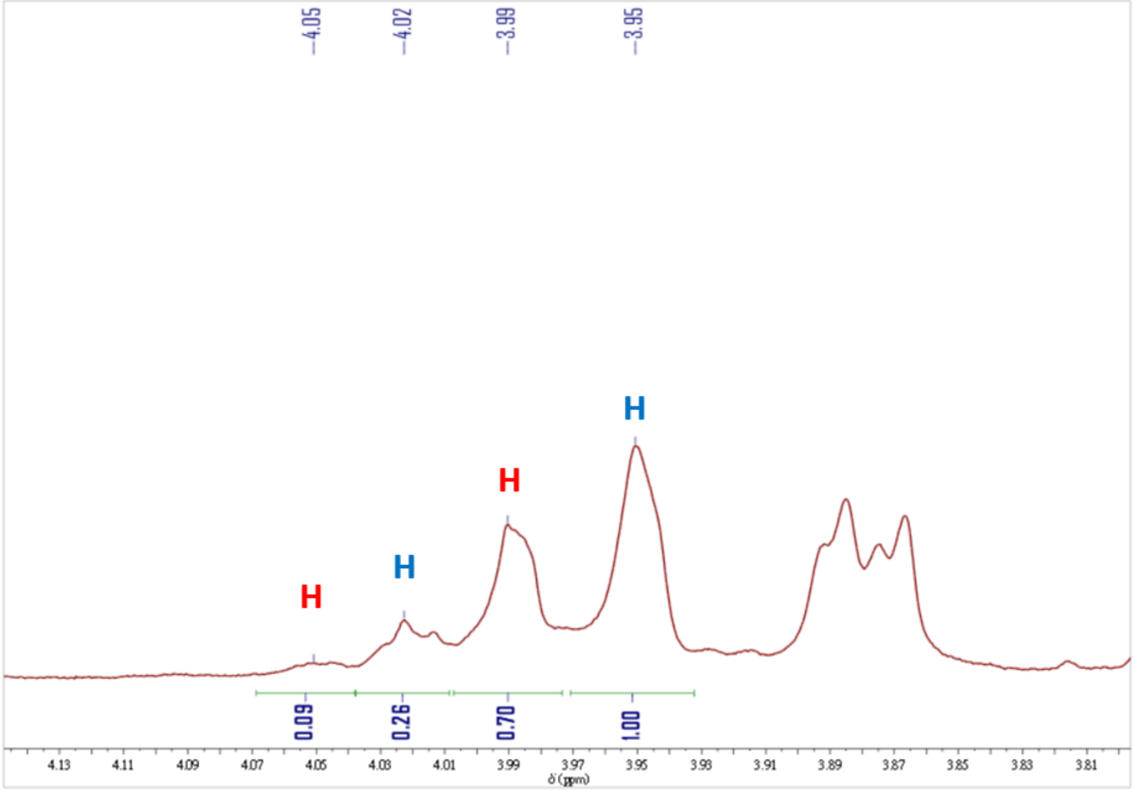

Supplementary Fig. 26: Partial ^{1}HNMR for degraded ball-milled **PN1**.

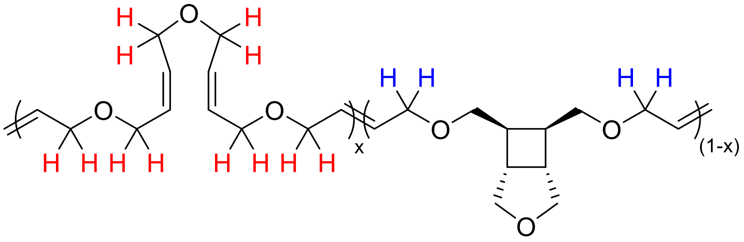

$$\frac{12x}{4(1-x)+12x} = \frac{0.09+0.7}{0.09+0.7+0.26+1}$$

$$12x = (8x+4) * 0.385$$

$$8.92x = 1.54$$

$$x = 17.2\%$$

VIII. Mass Spectrometry

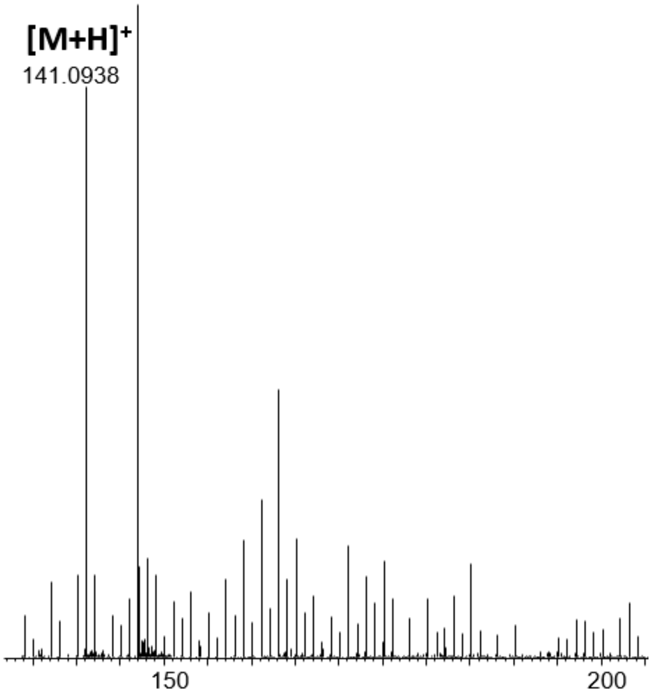

Supplementary Fig. 27: ASAP-MS spectrum for compound **DHF dimer**.

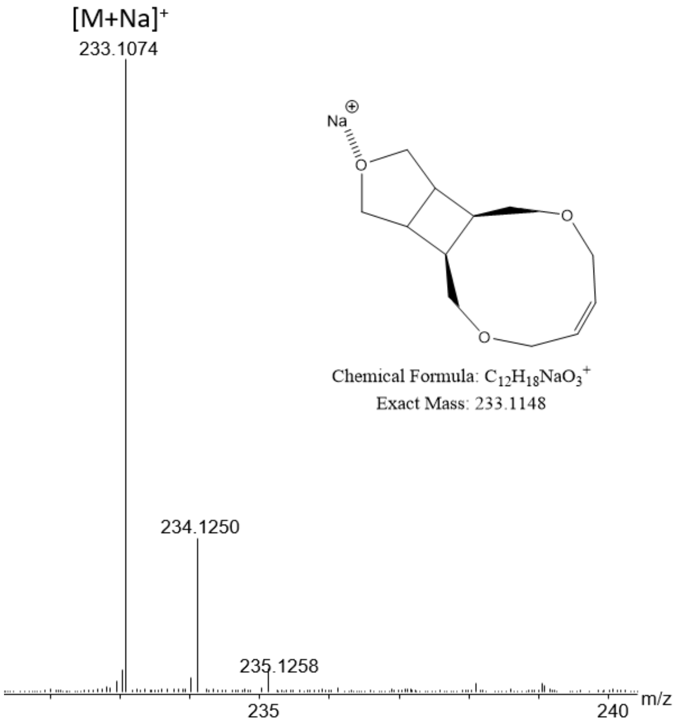

Supplementary Fig. 28: ESI-MS spectrum for compound **M1**.

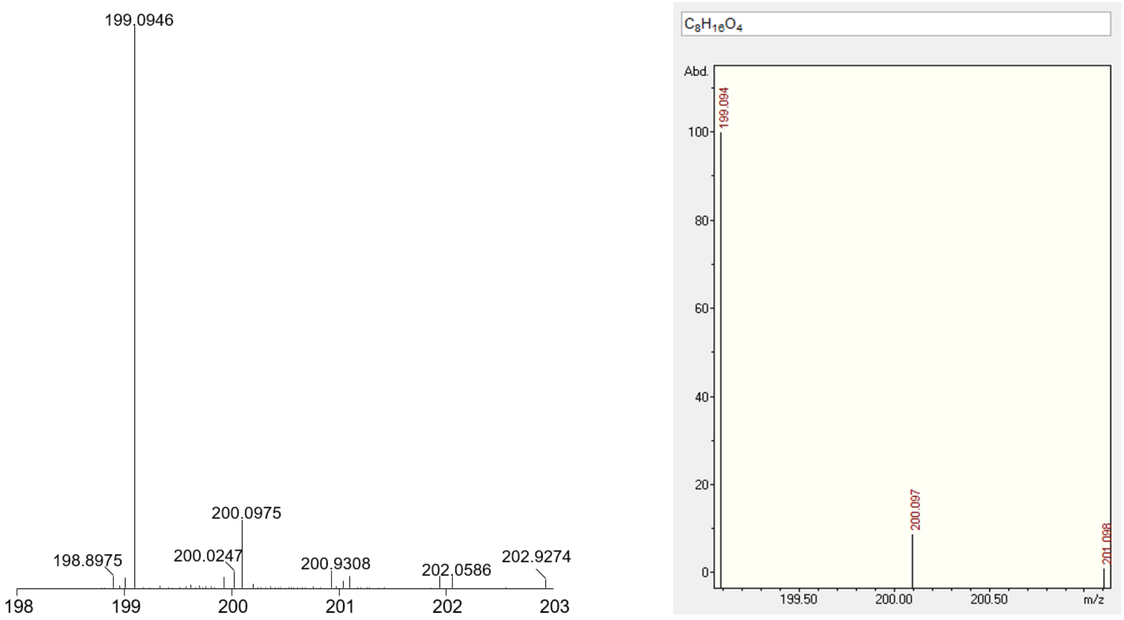

Supplementary Fig. 29: ESI-MS spectrum for compound **2**.

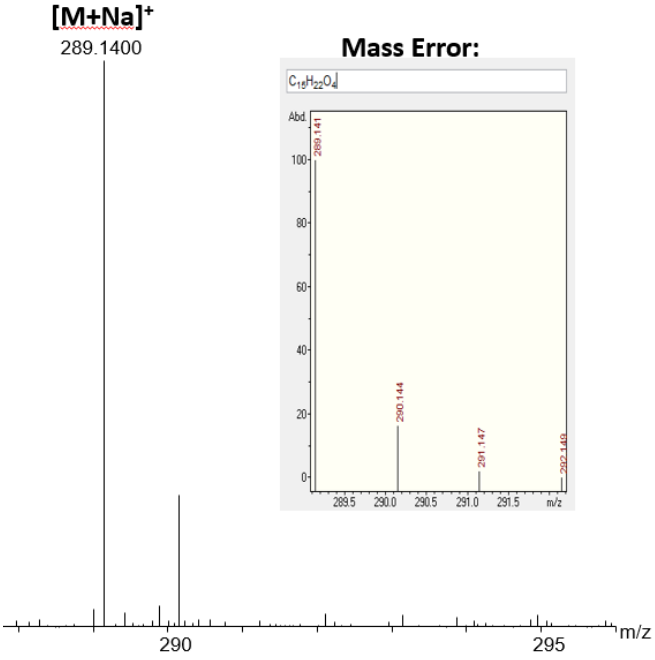

Supplementary Fig. 30: ESI-MS spectrum for compound **3**.

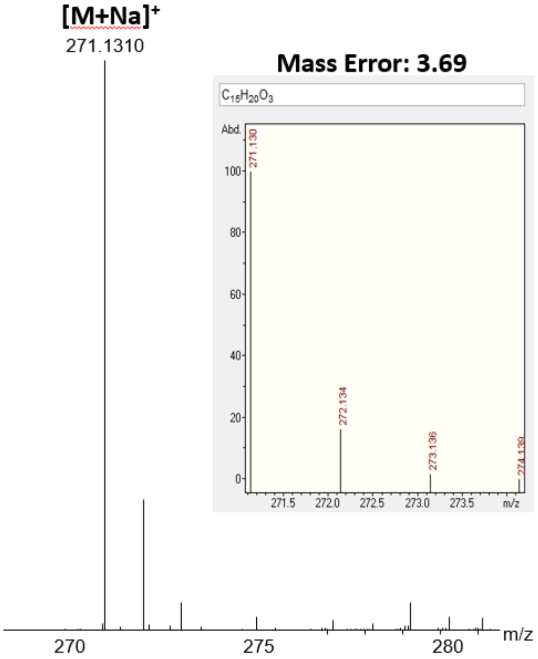

Supplementary Fig. 31: ESI-MS spectrum for compound **4**.

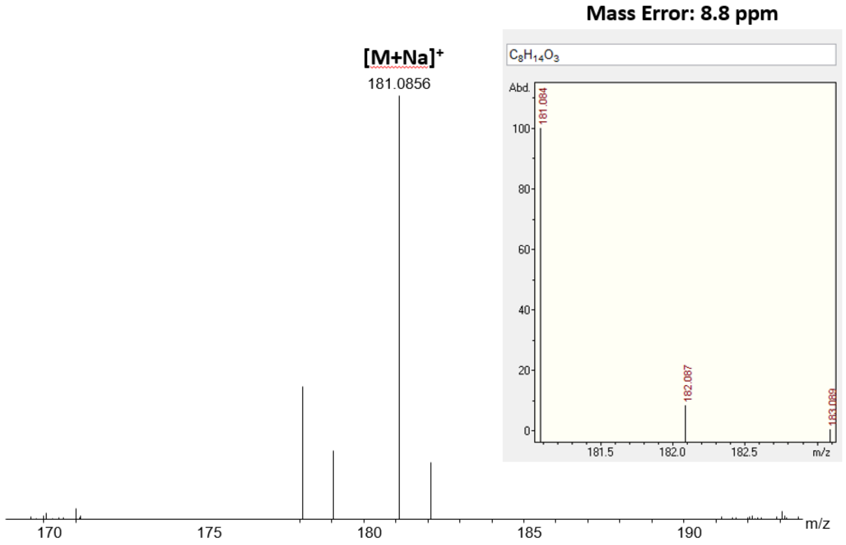

Supplementary Fig. 32: ESI-MS spectrum for compound **1**.

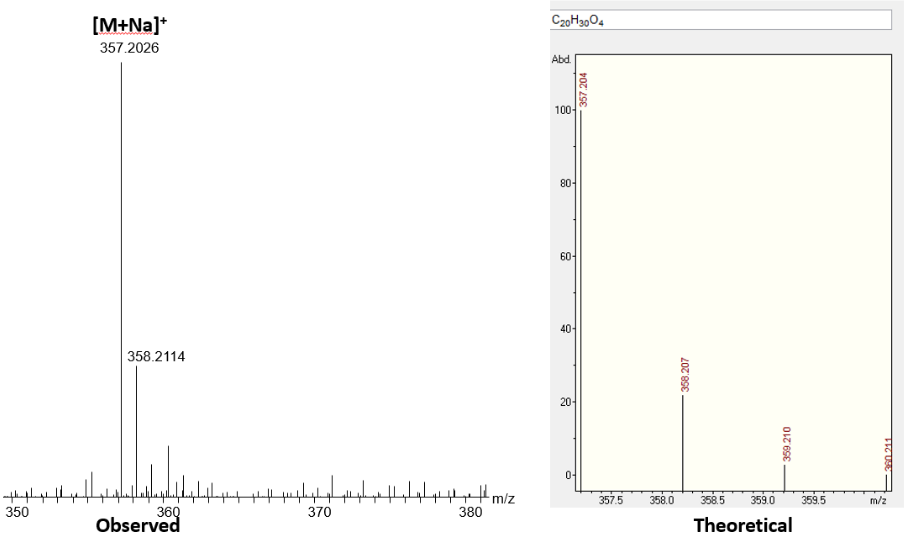

Supplementary Fig. 33: ESI-MS spectrum for bis-cyclooctene ester.

IX. X-Ray Crystallographic Study

X-Ray Crystallographic Study of M1

Single crystal of **M1** was obtained by making a saturated solution of **M1** with EA/Hexane (1:5) at room temperature. The solution was left for 1 day to afford transparent needle-like single crystals. X-ray intensity data were measured on a Bruker CCD-based and PHOTON II CPAD-based diffractometer with dual Cu/Mo ImuS microfocus optics (Mo Kα radiation, λ =0.71073 Å). Crystals were mounted on a cryoloop using Paratone oil and placed under a steam of nitrogen at 100 K (Oxford Cryosystems). The detector was placed at a distance of 5.00 cm from the crystal. The data were corrected for absorption and the structures were refined using the Bruker SHELXTL Software Package (Version 6.1) and were solved using direct methods until the final anisotropic full-matrix, least squares refinement of F^2 converged. CCDC 2179611 contains the supplementary crystallographic data for this work. These data can be obtained free of charge from The Cambridge Crystallographic Data Centre via www.ccdc.cam.ac.uk/structures.

Supplementary Table 4. Crystal data and structure refinement parameters.

**Compound**	**M1**
CCDC	2179611
Empirical formula	$C_{12}H_{18}O_3$
Formula weight	210.26
Crystal system	monoclinic
Space group	P2(1)/n
a/ Å	5.5345(4)
b/ Å	15.2895(11)
c/ Å	12.7056(10)
α(°)	90
β(°)	98.571(3)
γ(°)	90
Volume (Å^3)	1063.14(14)
Z	4
Dc (Mg/m^3)	1.314
μ (mm^{-1})	0.093
F(000)	456
reflns collected	2639
indep. reflns	2178
GOF on F^2	0.939
R1 (on F_o^2, $I > 2\sigma(I)$)	0.0437
wR2 (on F_o^2, $I > 2\sigma(I)$)	0.1361
R1 (all data)	0.0532
wR2 (all data)	0.1439

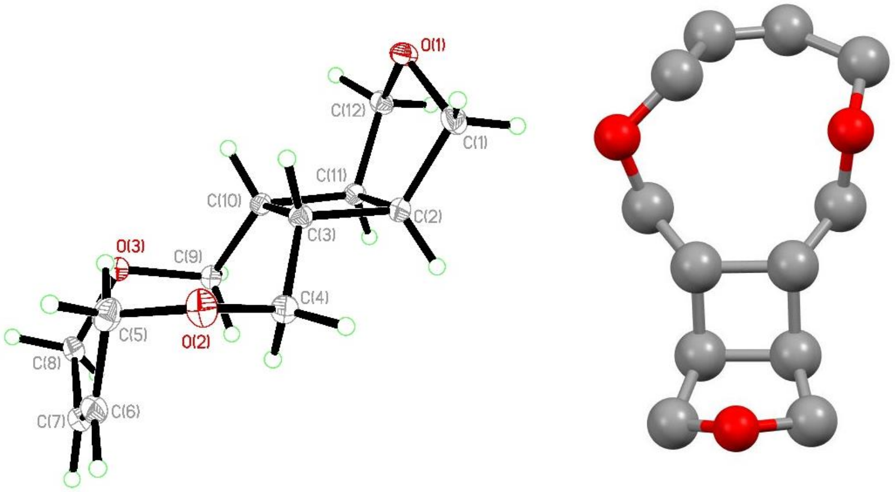

Supplementary Fig. 34: Left: Molecular structure of **M1** with 35% thermal ellipsoids. Right: Ball and stick diagram of **M1** orthogonal to the rings with hydrogen atom positions omitted for clarity.

X. ^{1}H and ^{13}C NMR Spectra

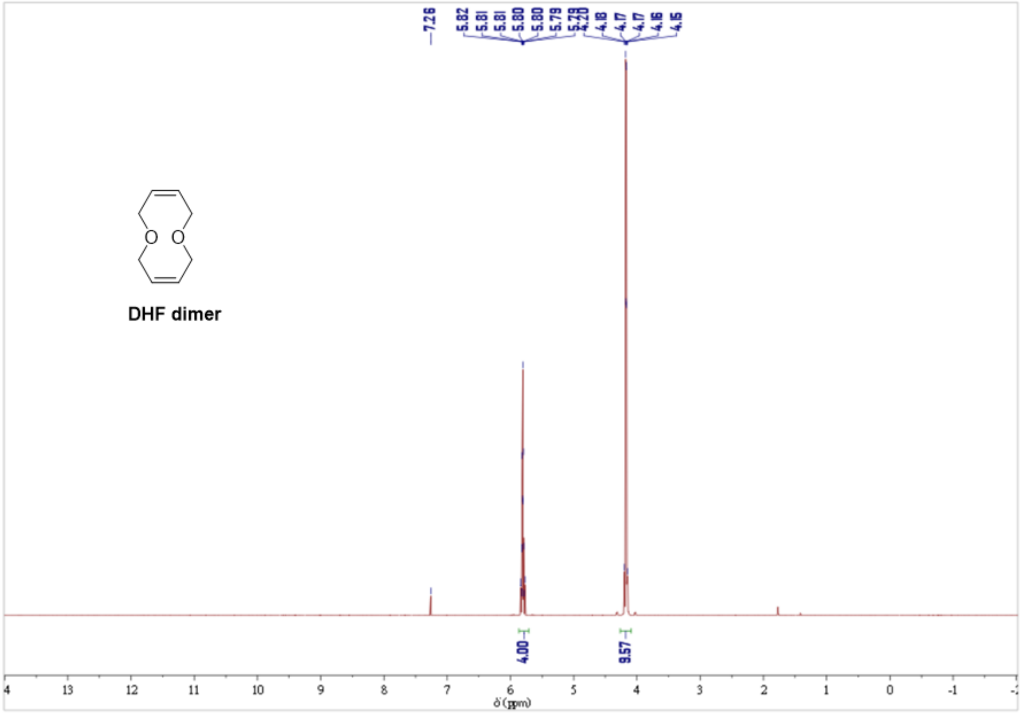

Supplementary Fig. 35: ^{1}H NMR spectrum for **DHF dimer**.

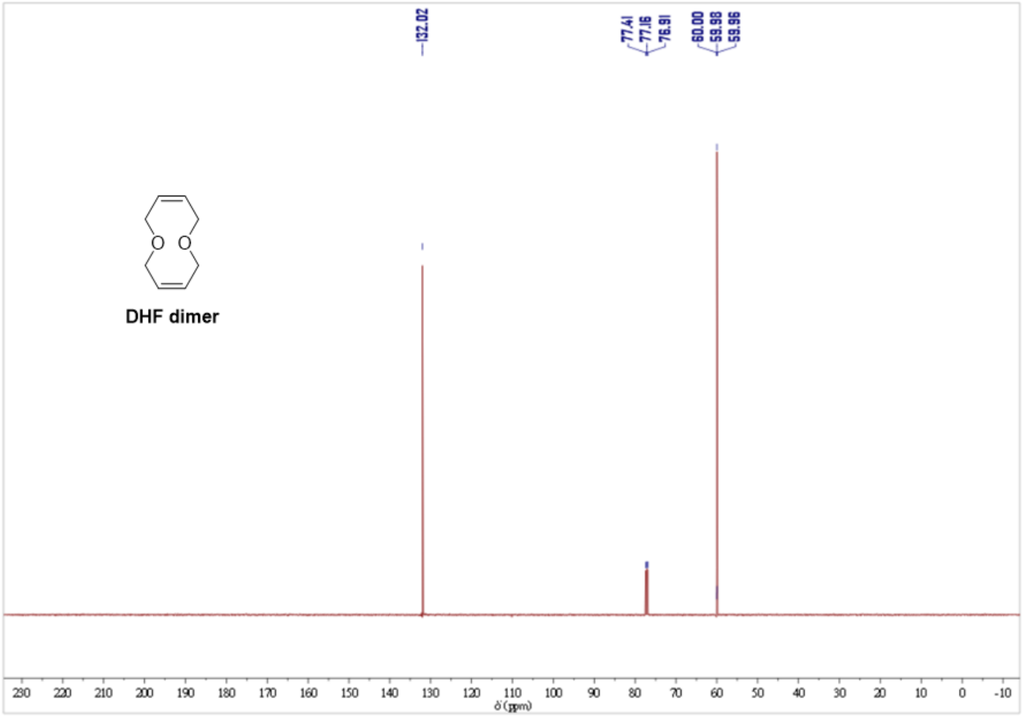

Supplementary Fig. 36: ^{13}C NMR spectrum for **DHF dimer**.

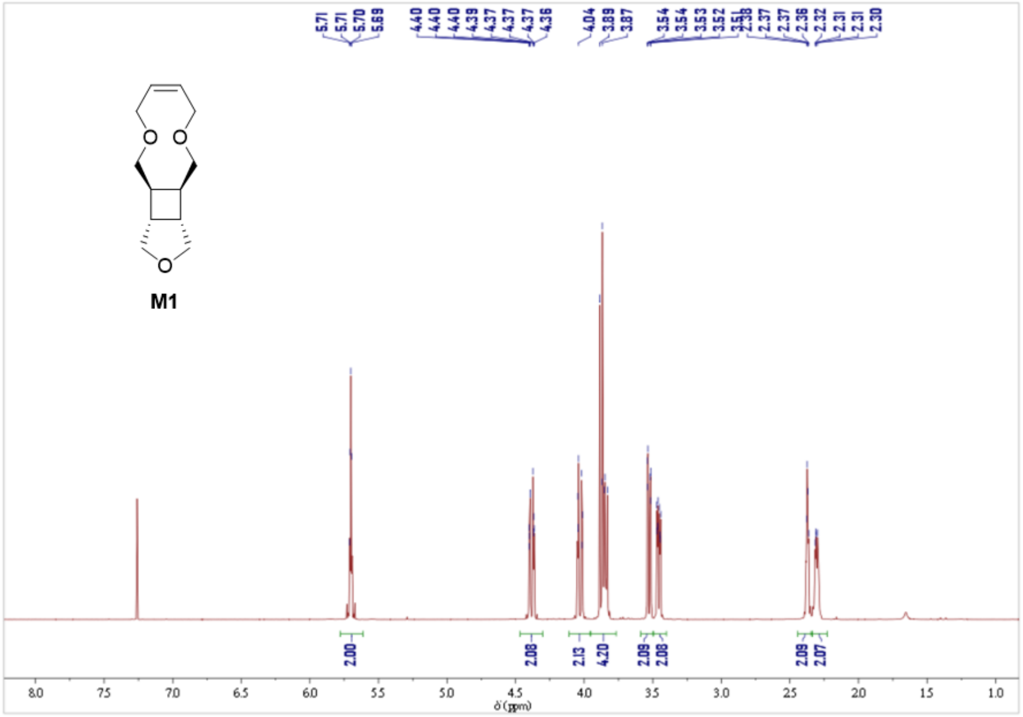

Supplementary Fig. 37: ^{1}H NMR spectrum for **M1**.

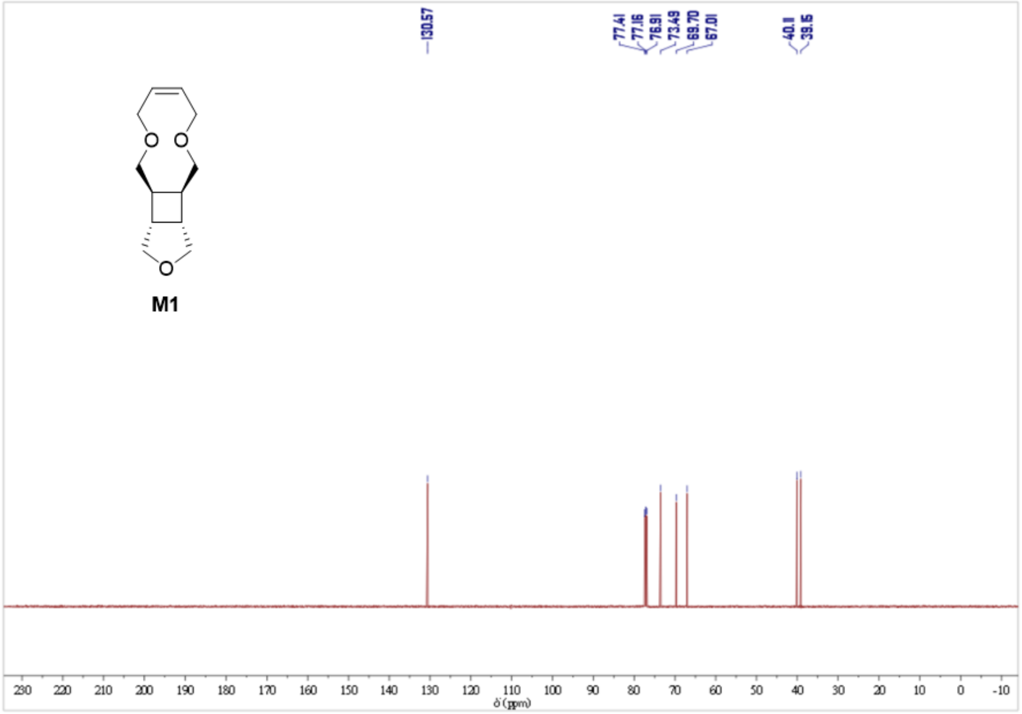

Supplementary Fig. 38: ^{13}C NMR spectrum for **M1**.

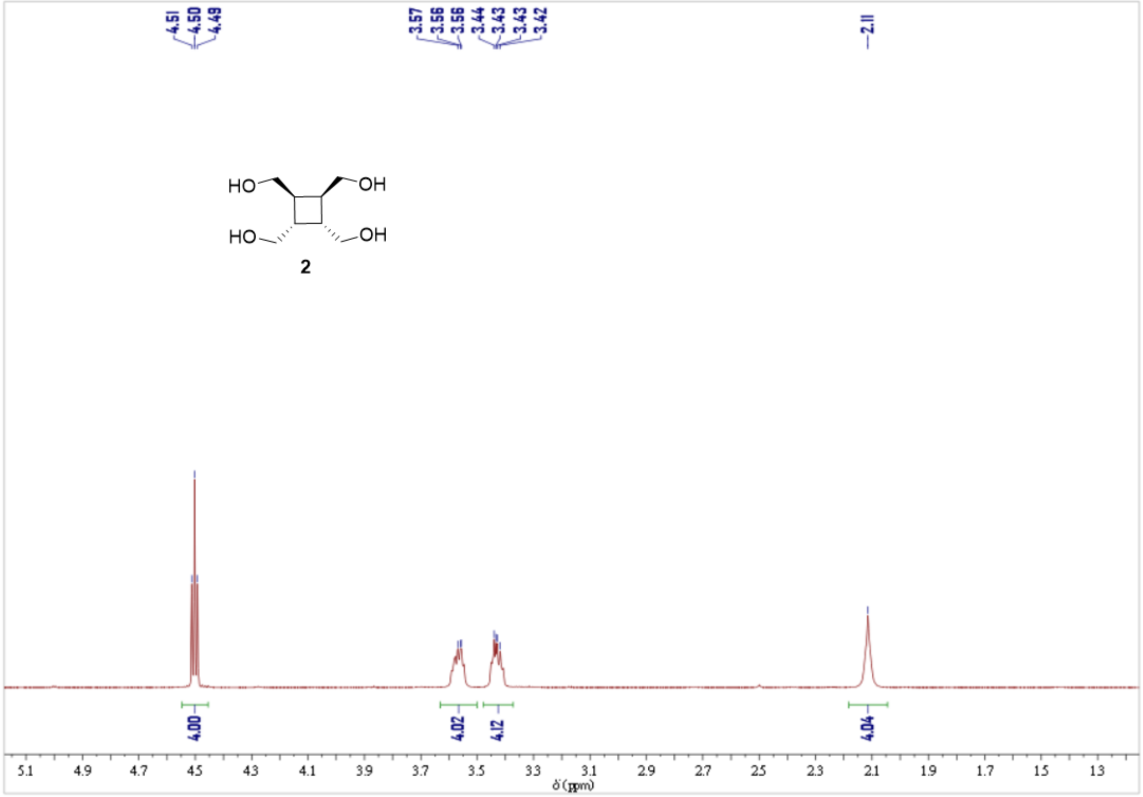

Supplementary Fig. 39: ^{1}H NMR spectrum for **2**.

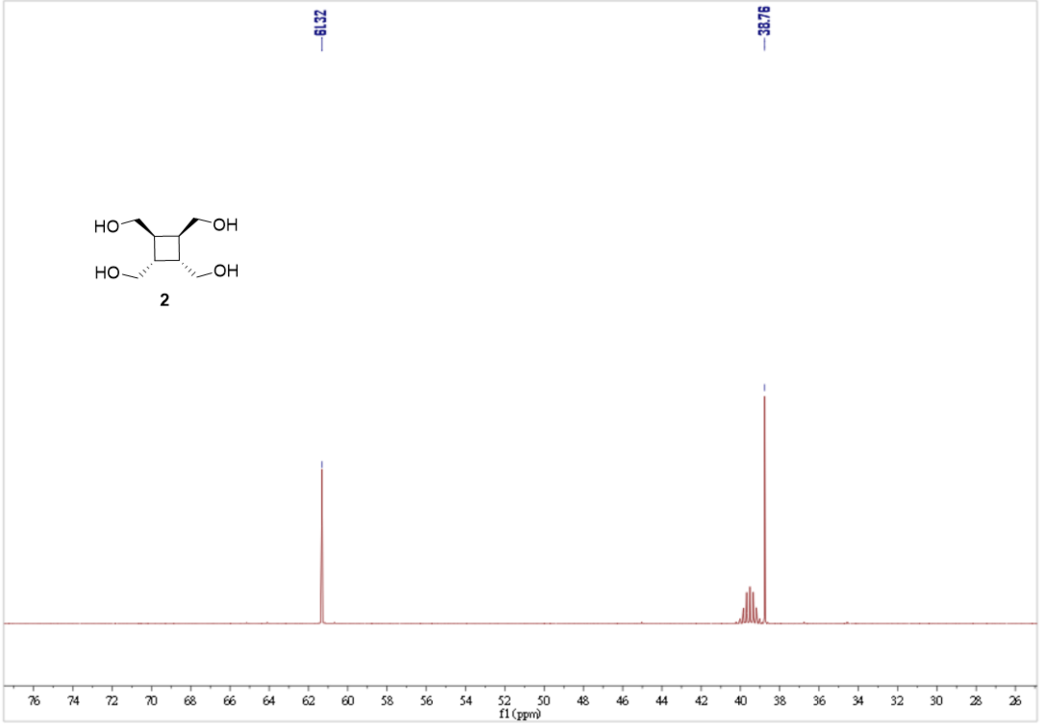

Supplementary Fig. 40: ^{13}C NMR spectrum for **2**.

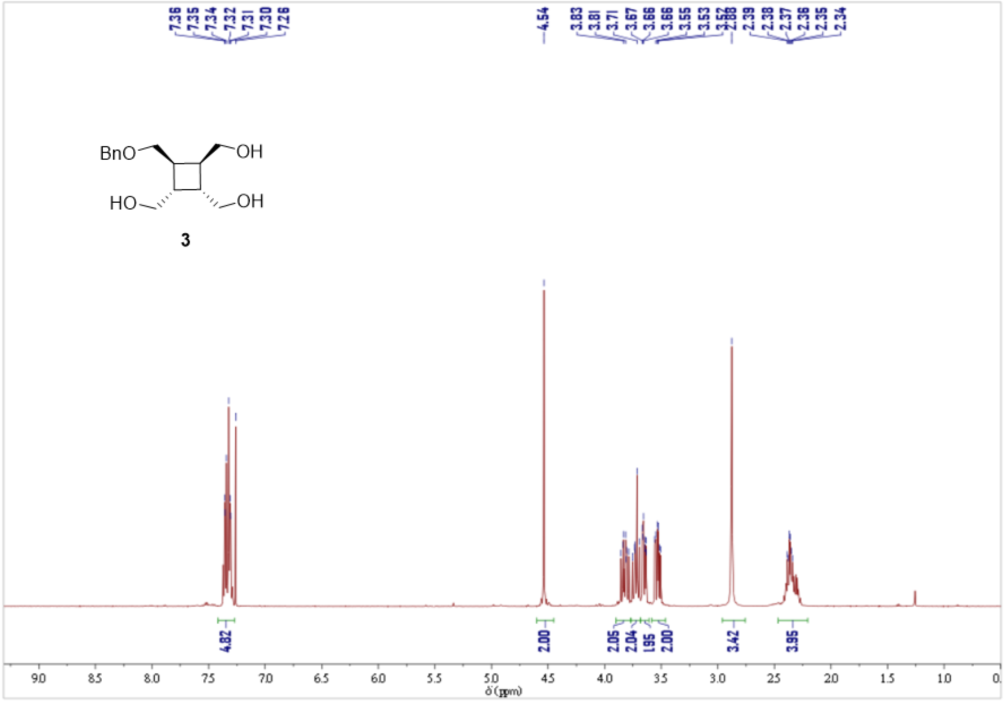

Supplementary Fig. 41: ^{1}H NMR spectrum for **3**.

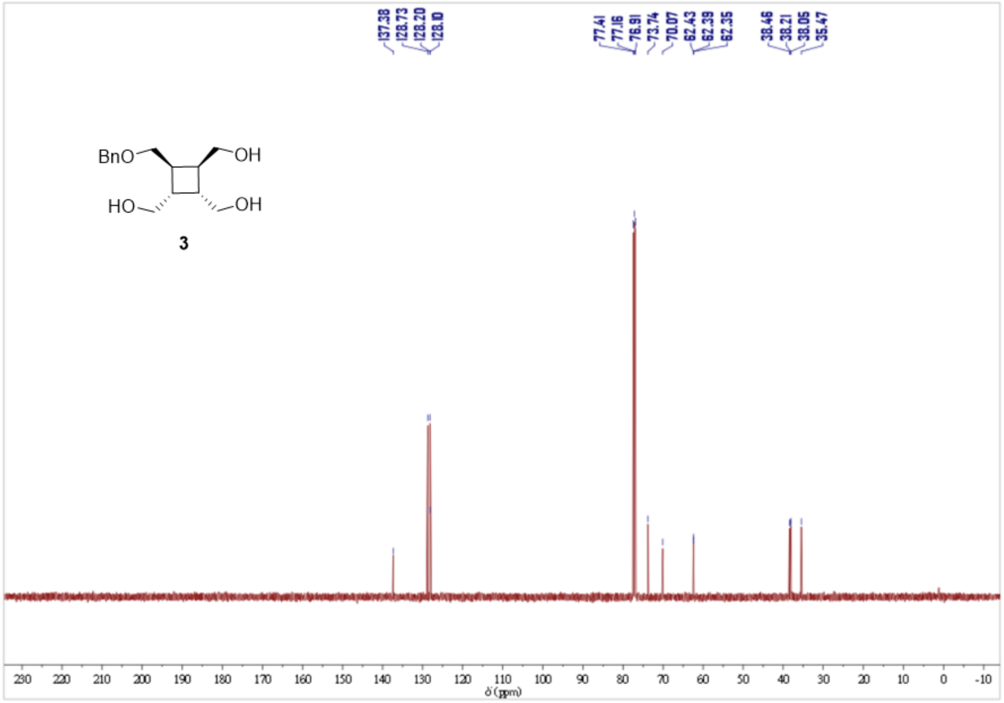

Supplementary Fig. 42: ^{13}C NMR spectrum for **3**.

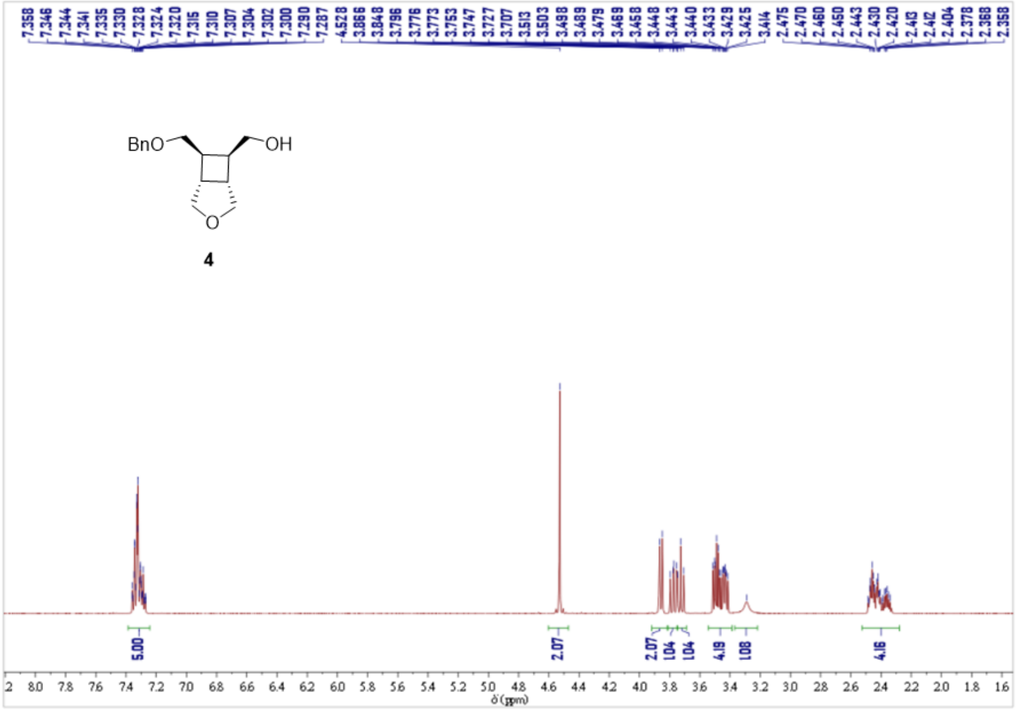

Supplementary Fig. 43: ^{1}H NMR spectrum for **4**.

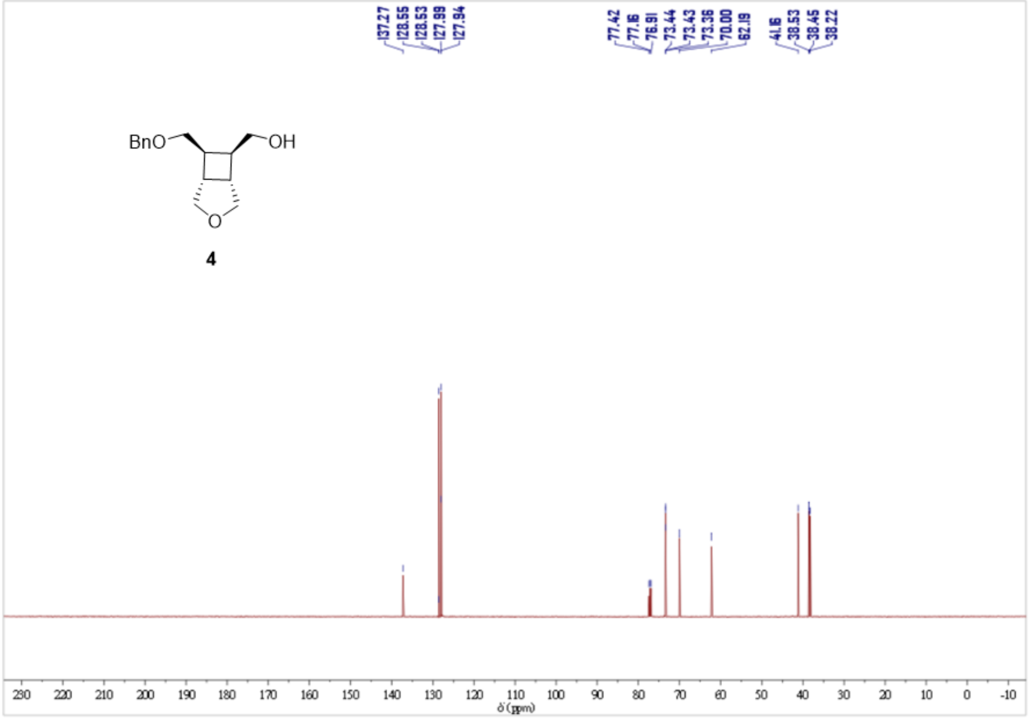

Supplementary Fig. 44: ^{13}C NMR spectrum for **4**.

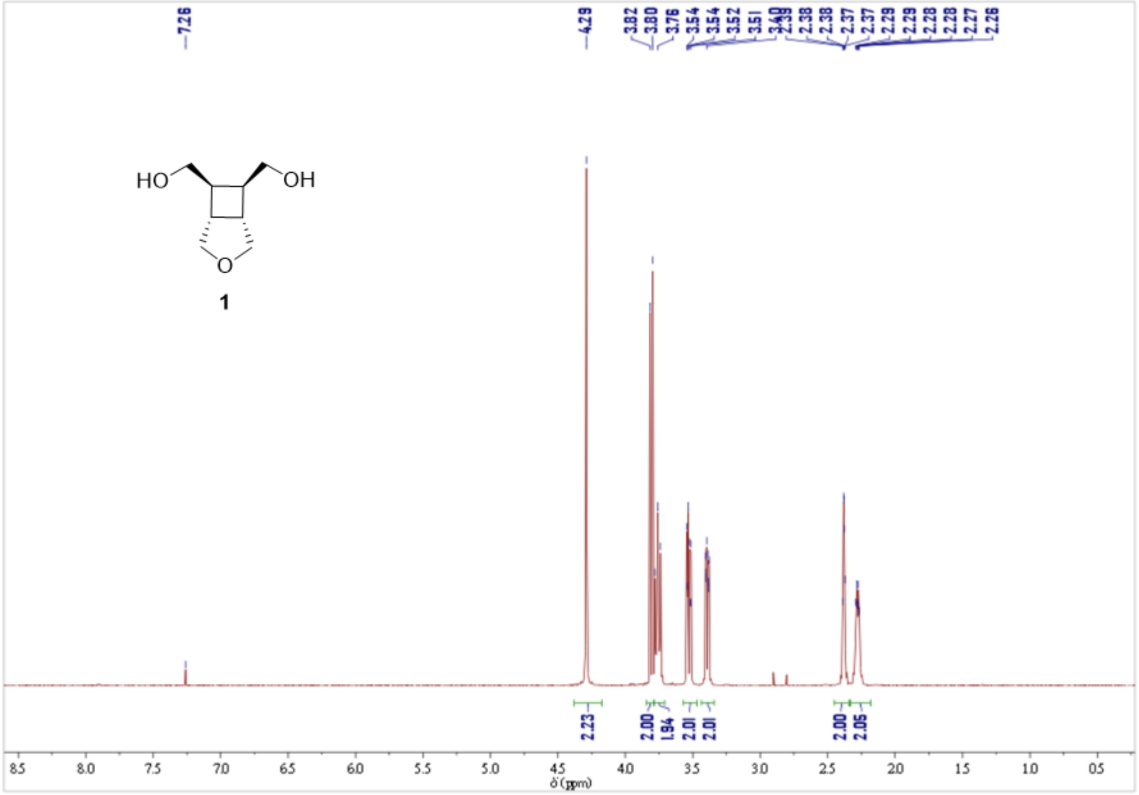

Supplementary Fig. 45: ^{1}H NMR spectrum for **1**.

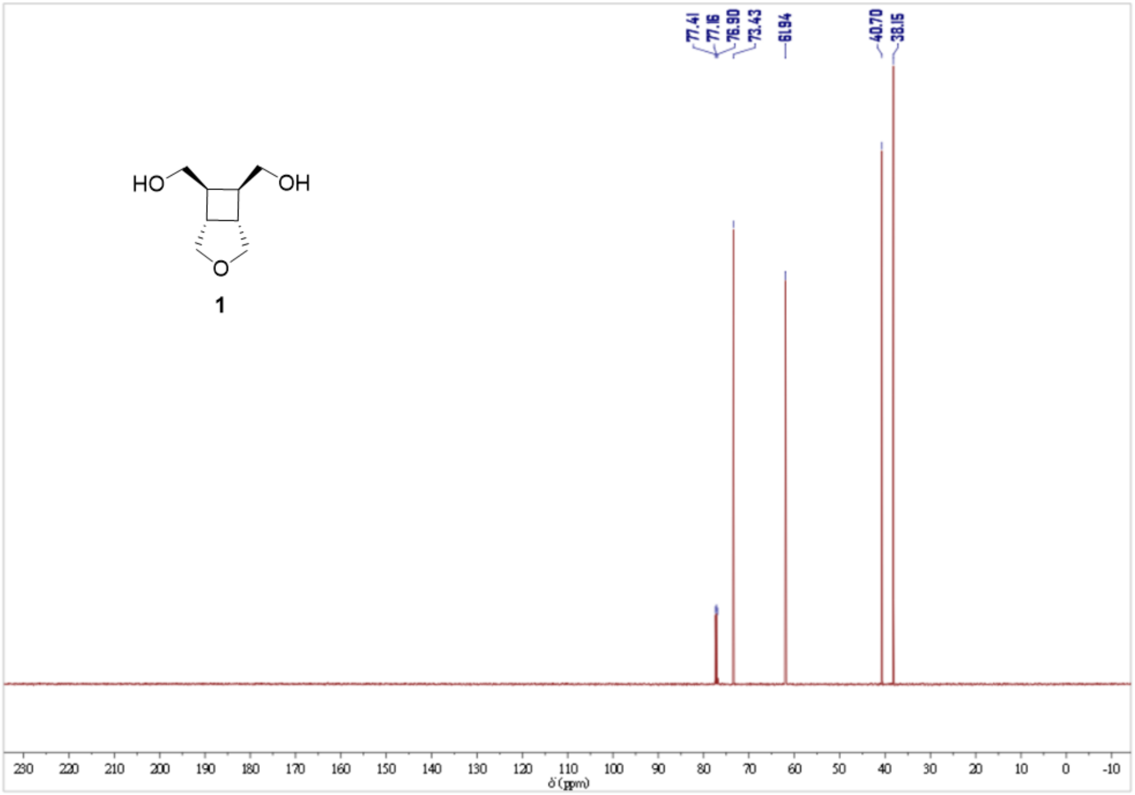

Supplementary Fig. 46: ^{13}C NMR spectrum for **1**.

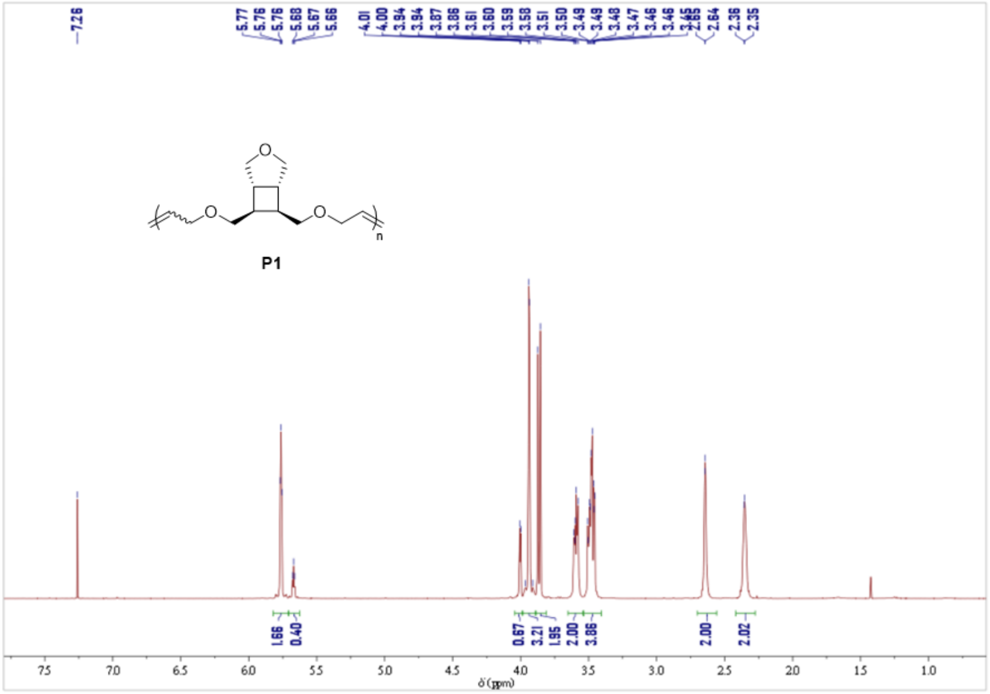

Supplementary Fig. 47: ^{1}H NMR spectrum for **P1**.

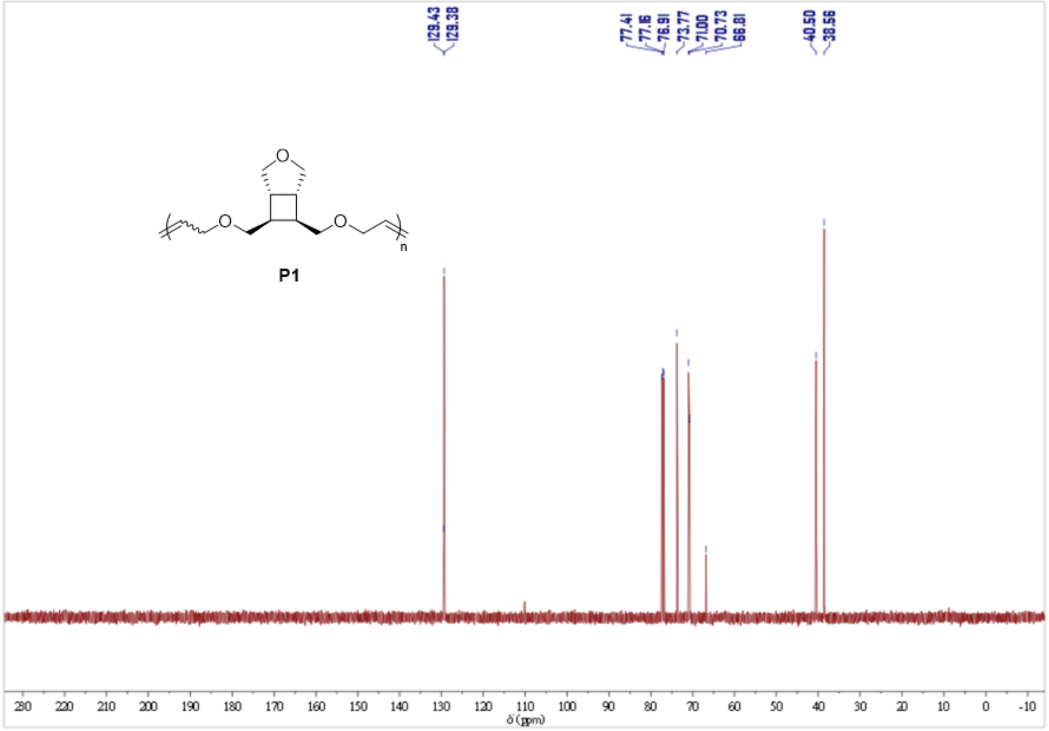

Supplementary Fig. 48: ^{13}C NMR spectrum for **P1**.

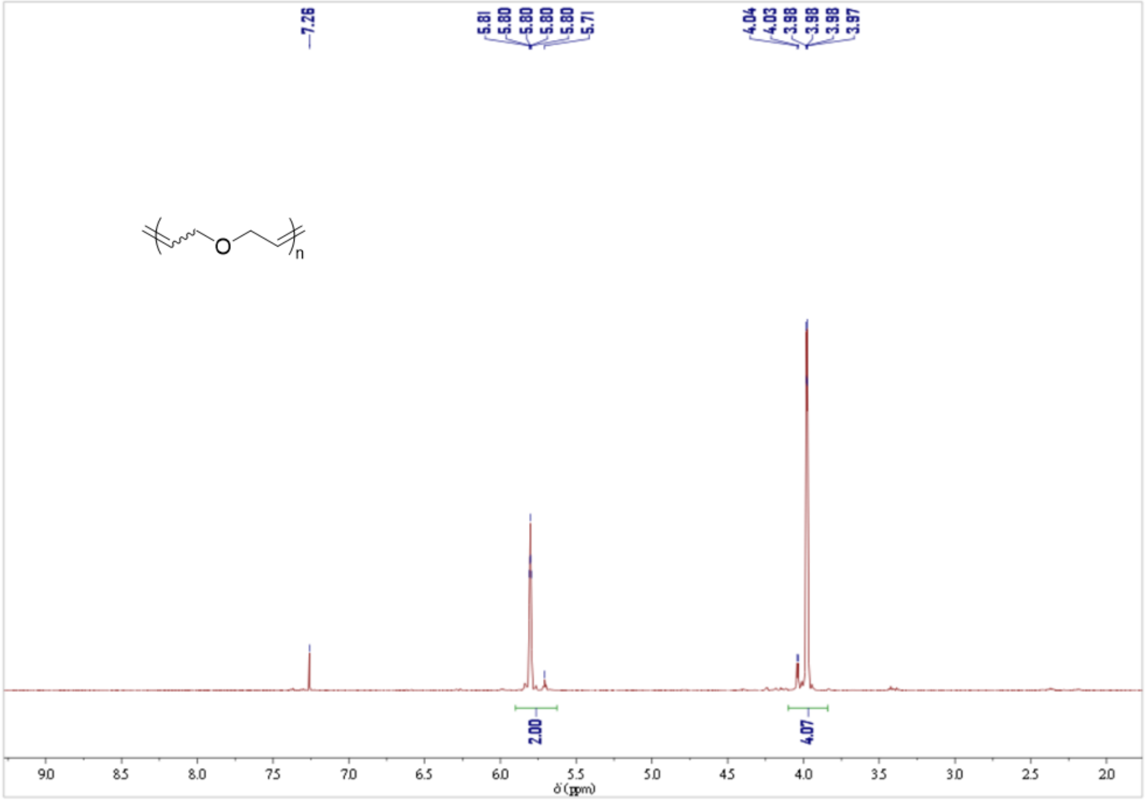

Supplementary Fig. 49: ^{1}H NMR spectrum for PDHF**.**

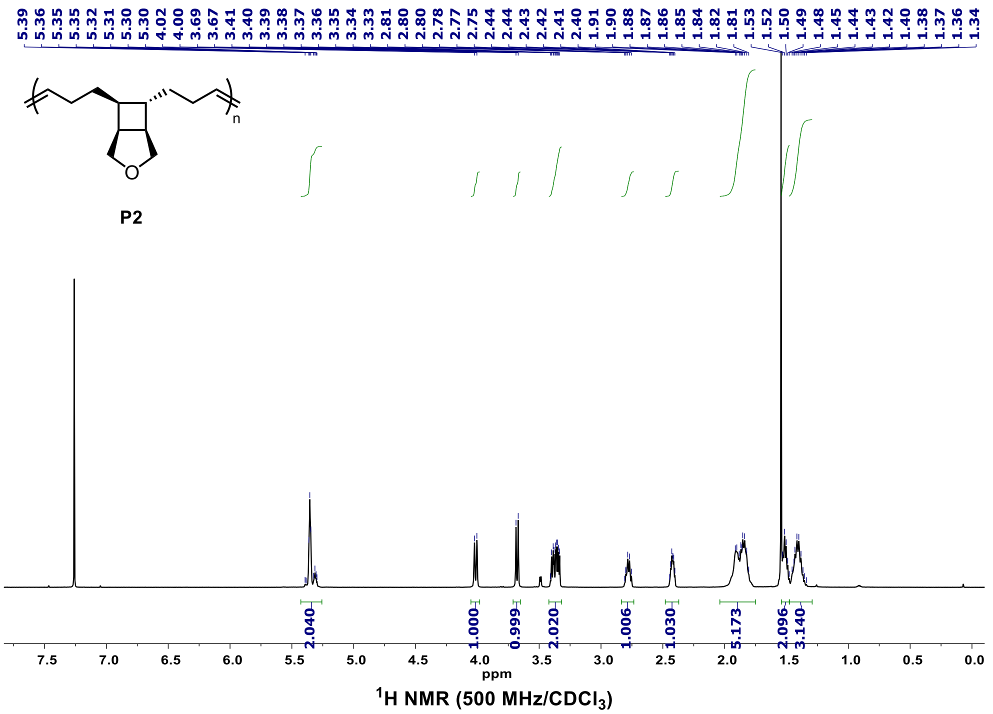

Supplementary Fig. 50: ^{1}HNMR of **P2.**

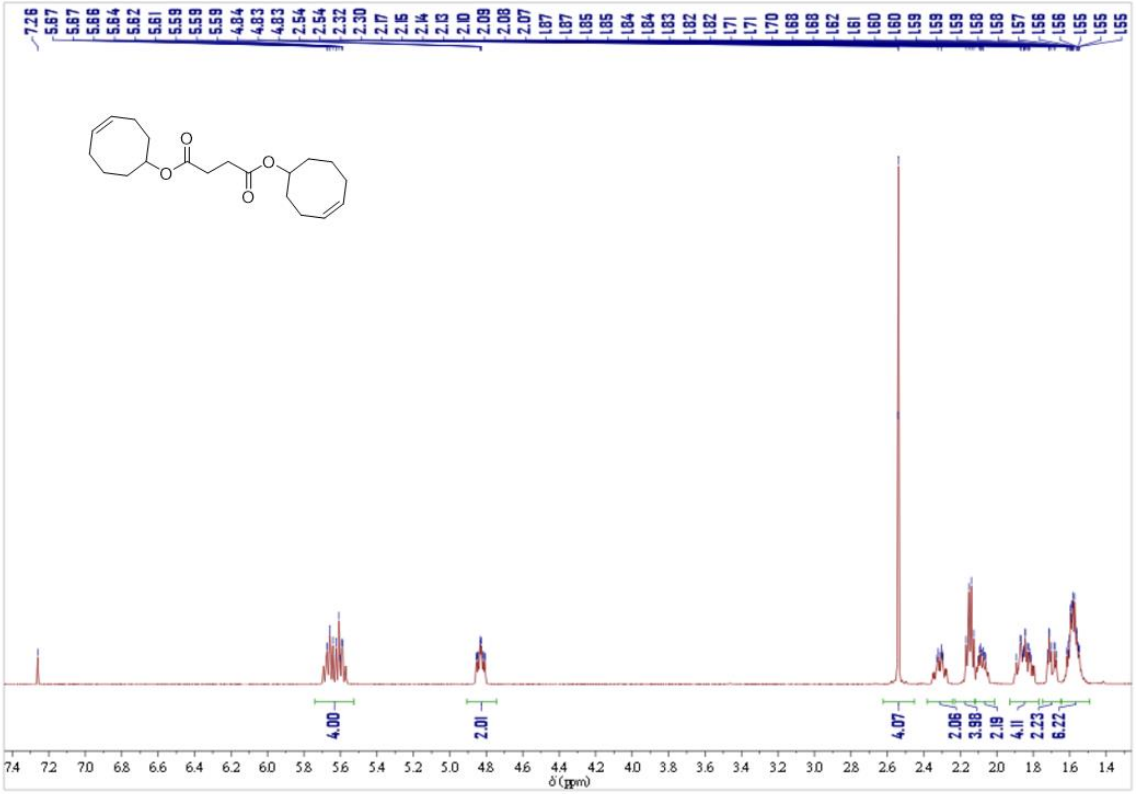

Supplementary Fig. 51: ^{1}HNMR of biscyclooctene ester.

51

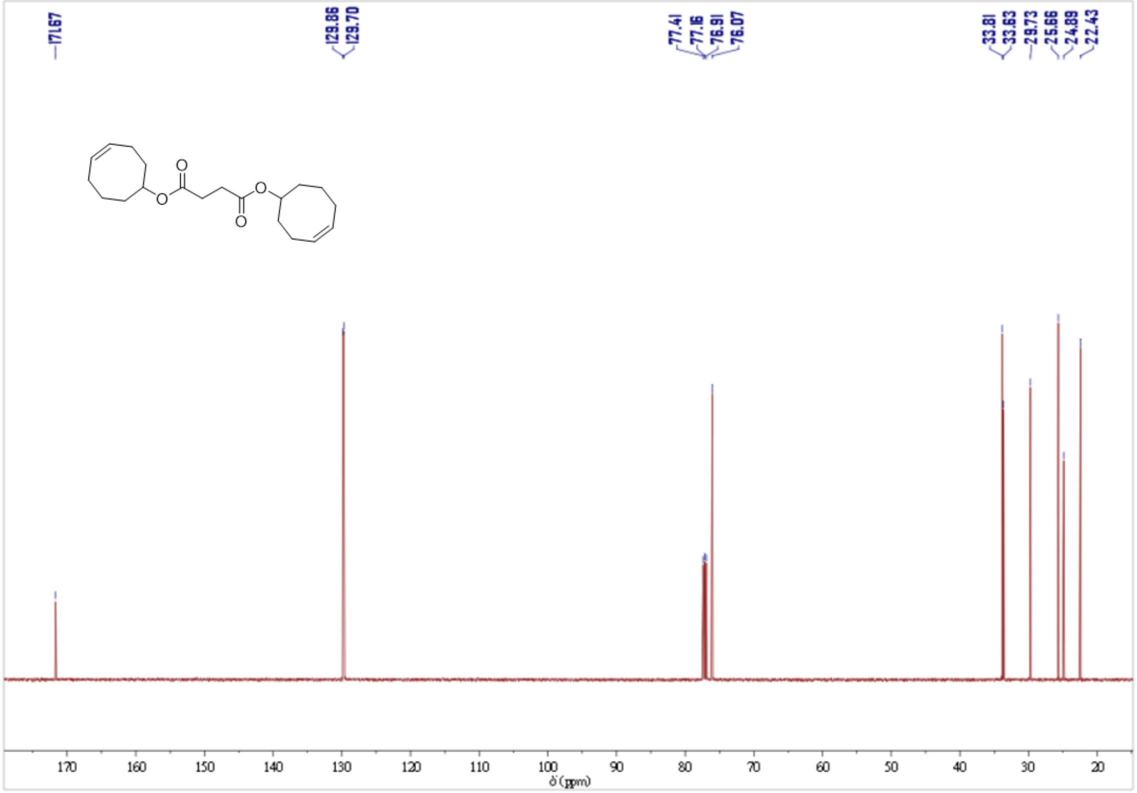

Supplementary Fig. 52: ^{13}C NMR spectrum for biscyclooctene ester.

XI. Optimized Geometries

Optimized geometries from DFT calculations at B3LYP/6-31g(d,p) level.

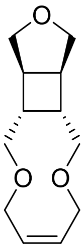

C 1.82361 -0.88345 0.64603
C 1.81376 0.68218 0.61001
C 3.27873 1.05566 0.40650
O 3.83313 -0.04102 -0.32341
C 3.26841 -1.22626 0.24418
O -1.64044 -1.16030 -0.85465
C -0.60278 -1.36564 0.10010
C -2.92441 -1.50701 -0.36033
C -3.48978 -0.59463 0.71258
C -3.30664 0.72493 0.83333
C -2.48702 1.57673 -0.10682
O -1.10310 1.49375 0.22909
C -0.20747 1.57144 -0.87244
C 0.96114 0.61852 -0.69039
C 0.73502 -0.91445 -0.46844
H 1.53925 -1.37304 1.58282
H 1.32669 1.18476 1.44787
H 3.43423 1.96052 -0.18945
H 3.79251 1.18010 1.37496
H 3.34856 -2.02138 -0.50335
H 3.84138 -1.52926 1.13586
H -0.53841 -2.44058 0.34756
H -0.82610 -0.81918 1.02307
H -3.58711 -1.50757 -1.23608
H -2.91463 -2.53966 0.02682
H -4.11755 -1.09461 1.45009
H -3.77210 1.23238 1.67602

H -2.63707 1.23123 -1.13704
H -2.82573 2.62291 -0.05365
H -0.72946 1.31893 -1.80525
H 0.17210 2.60243 -0.97625
H 1.62233 0.75366 -1.55513
H 0.97892 -1.52919 -1.34310

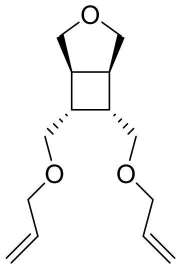

C 2.38906 0.68181 0.23723
C 2.20818 -0.28707 -0.98123
C 3.55778 -1.00226 -1.07522
O 4.11553 -0.93143 0.23823
C 3.81119 0.38020 0.71888
O -0.32383 1.67065 0.32574
C 0.04922 0.80606 1.39474
C -1.42247 2.50896 0.65203
C -1.68785 3.43084 -0.50028
C -3.34130 -2.68538 0.31443
C -2.45118 -1.96437 -0.65328
O -1.17383 -1.79093 -0.05930
C -0.23993 -1.16169 -0.93019
C 1.09163 -1.04974 -0.21089
C 1.23758 -0.05117 0.98686
C -2.88393 3.61620 -1.05573
C -4.57044 -2.29303 0.64423
H 2.20708 1.74219 0.05456
H 1.89187 0.16433 -1.92598
H 3.48737 -2.05930 -1.34948
H 4.21476 -0.49359 -1.80112
H 3.92381 0.36840 1.80746
H 4.52086 1.11482 0.30213

H -0.79680 0.16060 1.67285
H 0.31950 1.41211 2.27763
H -2.31882 1.91201 0.88590
H -1.18052 3.09661 1.55688
H -0.82071 3.97967 -0.86481
H -2.92156 -3.59629 0.73866
H -2.34929 -2.55613 -1.58211
H -2.89206 -0.99465 -0.93743
H -0.11086 -1.76563 -1.84669
H -0.60549 -0.16939 -1.22819
H 1.40989 -2.05746 0.07886
H 1.58570 -0.56446 1.89116
H -3.76213 3.07388 -0.71337
H -3.03364 4.32065 -1.86819
H -5.19046 -2.86809 1.32517
H -5.00679 -1.38287 0.23936

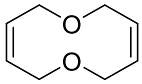

C 0.50826 0.21790 2.30930
C 1.53866 0.05505 1.47163
C -0.95249 -0.13526 2.14375
O -1.36389 -0.55086 0.85705
C -1.49258 0.53021 -0.08445
C -1.53866 -0.05505 -1.47163
C -0.50826 -0.21790 -2.30930
C 0.95249 0.13526 -2.14375
O 1.36389 0.55086 -0.85705
C 1.49258 -0.53021 0.08445
H 0.71799 0.67302 3.27876
H 2.51229 0.41511 1.79917
H -1.19602 -0.96558 2.82129
H -1.55495 0.72445 2.48841
H -2.42549 1.07673 0.12583
H -0.65448 1.22284 0.01361
H -2.51229 -0.41511 -1.79917

H -0.71799 -0.67302 -3.27876
H 1.55495 -0.72445 -2.48841
H 1.19602 0.96558 -2.82129
H 0.65448 -1.22284 -0.01361
H 2.42549 -1.07673 -0.12583

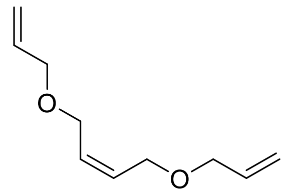

C 0.60753 -2.00316 -0.35206
C 1.90549 -1.68201 -0.31961
C 2.59647 -0.71044 0.60159
O 3.25762 0.3228 -0.13153
O -1.47361 -0.87682 -0.2435
C -0.47641 -1.50521 0.55807
C 2.36485 1.2446 -0.76641
C 1.66551 2.14878 0.21327
C -3.64747 0.05664 -0.43842
C -2.64405 -0.5491 0.49573
C -4.28538 1.20414 -0.21492
C 0.34023 2.22233 0.34691
H 0.26063 -2.70013 -1.11366
H 2.56478 -2.13102 -1.06184
H 1.9061 -0.28326 1.33895
H 3.3968 -1.21883 1.15142
H -0.0967 -0.80338 1.31333
H -0.92275 -2.35745 1.10145
H 3.00609 1.83211 -1.43342
H 1.6264 0.71558 -1.3841
H 2.31504 2.76481 0.83496
H -3.85 -0.52053 -1.33924
H -2.4108 0.14731 1.31751
H -3.05728 -1.46633 0.95437
H -5.02849 1.58838 -0.90684
H -4.09234 1.80112 0.67326

H -0.12311 2.90419 1.05502
H -0.32707 1.60021 -0.24588

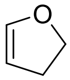

C -1.20139 -0.06956 0.00737
C -0.57230 1.10565 0.03694
C 0.91858 0.85610 -0.05116
C 0.97279 -0.68948 0.05916
O -0.39324 -1.17171 -0.05730
H -2.26097 -0.29389 0.01649
H -1.04378 2.07771 0.06947
H 1.33864 1.21905 -0.99915
H 1.48702 1.33723 0.75307
H 1.56223 -1.16661 -0.72768
H 1.35672 -1.01611 1.03234

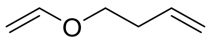

C 2.19889 -0.60745 -0.17063
C -2.00354 -0.44075 -0.07942
C -1.19023 0.74127 -0.53351
C 0.24114 0.73332 0.00145
O 0.90675 -0.40853 -0.53853
C 2.95552 0.13674 0.64125
C -3.15820 -0.36080 0.58109
H 2.58103 -1.50611 -0.64706
H -1.58677 -1.41745 -0.31939
H -1.13597 0.75945 -1.63060
H -1.67112 1.67483 -0.21847
H 0.77399 1.64588 -0.30067
H 0.24875 0.68054 1.09877
H 2.60623 1.03669 1.13148
H 3.97781 -0.16807 0.82791
H -3.70494 -1.24847 0.88496
H -3.60439 0.59700 0.83994

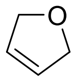

C 0.00000 1.17241 0.37105
C 0.00000 0.66529 -1.04492
C 0.00000 -0.66529 -1.04492
C 0.00000 -1.17241 0.37105
O 0.00000 0.00000 1.18998
H -0.88592 1.78774 0.59766
H 0.88592 1.78774 0.59766
H 0.00000 1.31618 -1.91197
H 0.00000 -1.31618 -1.91197
H -0.88592 -1.78774 0.59766
H 0.88592 -1.78774 0.59766

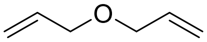

C 1.17134 -0.58121 0.15862
C 2.33689 0.32987 0.40191
C -2.33689 0.32987 -0.40191
C -1.17134 -0.58121 -0.15862
O 0.00000 0.20591 0.00000
C 3.49027 0.26926 -0.26078
C -3.49027 0.26926 0.26078
H 1.04502 -1.26584 1.01789
H 1.34851 -1.20969 -0.73036
H 2.19410 1.06231 1.19484
H -2.19410 1.06231 -1.19484
H -1.34851 -1.20969 0.73036
H -1.04502 -1.26584 -1.01789
H 3.65106 -0.44737 -1.06273
H 4.31935 0.92947 -0.02528
H -4.31935 0.92947 0.02528
H -3.65106 -0.44737 1.06273

XII. Supplementary References

1 Sathe, D. *et al.* Olefin metathesis–based chemically recyclable polymers enabled by fused-ring monomers. *Nat. Chem.* **13**, 743-750, (2021).

2 Boswell, B. R. *et al.* Mechanochemical synthesis of an elusive fluorinated polyacetylene. *Nat. Chem.* **13**, 41-46, (2021).

3 Breitenkamp, K., Simeone, J., Jin, E. & Emrick, T. Novel amphiphilic graft copolymers prepared by ring-opening metathesis polymerization of poly(ethylene glycol)-substituted cyclooctene macromonomers. *Macromolecules* **35**, 9249-9252, (2002).

4 Berkowski, K. L., Potisek, S. L., Hickenboth, C. R. & Moore, J. S. Ultrasound-induced site-specific cleavage of azo-functionalized poly(ethylene glycol). *Macromolecules* **38**, 8975-8978, (2005).

5 Lenhardt, J. M., Black Ramirez, A. L., Lee, B., Kouznetsova, T. B. & Craig, S. L. Mechanistic insights into the sonochemical activation of multimechanophore cyclopropanated polybutadiene polymers. *Macromolecules* **48**, 6396-6403, (2015).

6 Hsu, T.-G. *et al.* A polymer with "locked" degradability: Superior backbone stability and accessible degradability enabled by mechanophore installation. *J. Am. Chem. Soc.* **142**, 2100-2104, (2020).
