# Supplementary material for: Mechanochemically accessing a challenging-to-synthesize depolymerizable polymer
Source: Nat Commun. 2023 Jan 14;14:225. doi: 10.1038/s41467-023-35925-2 (PMC9840636; doi:10.1038/s41467-023-35925-2)
Supplement: Supplementary file 3 — Description of Additional Supplementary Files [file 41467_2023_35925_MOESM3_ESM.pdf]

File Name: Supplementary Data 1

Description: CIF file and checkcif file for M1
